# Development of Plastid Genomic Resources for Discrimination and Classification of *Epimedium wushanense* (Berberidaceae)

**DOI:** 10.3390/ijms20164003

**Published:** 2019-08-16

**Authors:** Mengyue Guo, Li Ren, Yanqin Xu, Baosheng Liao, Jingyuan Song, Ying Li, Nitin Mantri, Baolin Guo, Shilin Chen, Xiaohui Pang

**Affiliations:** 1 Key Lab of Chinese Medicine Resources Conservation, State Administration of Traditional Chinese Medicine of the People’s Republic of China, Institute of Medicinal Plant Development, Chinese Academy of Medical Sciences & Peking Union Medical College, Beijing 100193, China; 2 College of Pharmacy, Jiangxi University of Traditional Chinese Medicine, Nanchang 330004, China; 3 Institute of Chinese Materia Medica, China Academy of Chinese Medical Sciences, Beijing 100700, China; 4 Engineering Research Center of Tradition Chinese Medicine Resource, Ministry of Education, Institute of Medicinal Plant Development, Chinese Academy of Medical Sciences & Peking Union Medical College, Beijing 100193, China; 5 The Pangenomics Group, School of Science, RMIT University, Melbourne, VIC 3083, Australia

**Keywords:** plastid genome, *Epimedium*, phylogenetic analysis, identification

## Abstract

*Epimedium wushanense* (Berberidaceae) is recorded as the source plant of Epimedii Wushanensis Folium in the Chinese Pharmacopoeia. However, controversies exist on the classification of *E. wushanense* and its closely related species, namely, *E*. *pseudowushanense*, *E. chlorandrum*, *E. mikinorii*, *E. ilicifolium*, and *E. borealiguizhouense*. These species are often confused with one another because of their highly similar morphological characteristics. This confusion leads to misuse in the medicinal market threatening efficiency and safety. Here, we studied the plastid genomes of these *Epimedium* species. Results show that the plastid genomes of *E. wushanense* and its relative species are typical circular tetramerous structure, with lengths of 156,855–158,251 bp. A total of 112 genes were identified from the *Epimedium* plastid genomes, including 78 protein-coding, 30 tRNA, and 4 rRNA genes. A loss of *rpl32* gene in *E. chlorandrum* was found for the first time in this study. The phylogenetic trees constructed indicated that *E. wushanense* can be distinguished from its closely related species. *E. wushanense* shows a closer relationship to species in ser. *Dolichocerae*. In conclusion, the use of plastid genomes contributes useful genetic information for identifying medicinally important species *E. wushanense* and provides new evidence for understanding phylogenetic relationships within the *Epimedium* genus.

## 1. Introduction

The classification and identification of the genus *Epimedium* L. (Berberidaceae) has always been a research hotspot. Stearn [1] proposed the most comprehensive classification system for this genus, which was supported by geographical distribution and C-banding of chromosomes. In Stearn’s monograph, genus *Epimedium* is divided into subgenera *Rhizophyllum* and *Epimedium*. Furthermore, subgenus *Epimedium* is divided into four sections, namely, *Epimedium*, *Polyphyllon*, *Macroceras*, and *Diphyllon*. The section *Diphyllon*, consisting of approximately 47 known species in China, is further subdivided into four series, namely, *Campanulatae* Stearn, *Davidianae* Stearn, *Dolichocerae* Stearn, and *Brachycerae* Stearn. However, controversies still exist in the classification and discrimination of *Epimedium* species. On the one hand, phylogenetic research based on molecular markers [2], flavonoid types [3], and amplified fragment length polymorphisms (AFLP) [4] could not support Stearn’s classification system. On the other hand, the majority of *Epimedium* species are easily confused with one another due to their highly similar morphology, thereby complicating the distinction between these species. *E. wushanense* T.S. Ying and its closely related species is a typical example of species complex. Ying et al. [5] listed the distribution of *E. wushanense* in Chongqing, Guizhou, Hubei, and Sichuan in the Flora of China. On the contrary, Guo et al. [6] concluded that *E. wushanense* from Guangxi and Guizhou should be recognized as a new species *E*. *pseudowushanense* B.L. Guo. Moreover, *E. wushanense* is easily confused with *E. chlorandrum* Stearn, *E. mikinorii* Stearn, *E. ilicifolium* Stearn, and *E. borealiguizhouense* S.Z. He & Y.K. Yang due to their highly similar morphological characteristics [7]. The dried leaves of *E. wushanense* are used as Epimedii Wushanensis Folium (Wushan Yinyanghuo) in the Chinese Pharmacopeia [8]. Considering differences in bioactive constituents among *Epimedium* species [9], incorrect identification of species is likely to result in potential risks in herbal efficacy and medical safety. However, only a few reports on the taxonomic analysis and authentication of *E. wushanense* and its related species are available [10,11]. Therefore, a systematic classification and comprehensive identification of *E. wushanense* and its closely related species is urgently needed.

The complete plastid genome, containing variant genetic information, has been extensively utilized in plant identification and phylogenetic studies. Most plastid genomes in angiosperms are circular DNA molecules ranging from 120 kb to 160 kb in length [12]. They have the advantages of maternal inheritance, single structure, and high conservation of gene content and genome structure [13,14]. Jose et al. [15] performed a comprehensive study on the evolution and variability of the genus *Citrus* to provide a good understanding of its phylogenetic relationships. Curci et al. [16] developed plastid resources for the genus *Cynara* and found that plastid genome can be used as a reliable and valuable molecular marker for *Cynara* identification. In addition, Pessoa-Filho et al. [17] investigated the phylogenetic relationships of four *Urochloa* species on the basis of their plastid genomes. Therefore, plastid genomes were expected to be an effective resource for discriminating *E. wushanense* and its related species. 

Here, we studied the plastid genomes of these *Epimedium* species. We successfully discriminate them based on plastid genome sequence and provide useful taxonomic information for the genus *Epimedium*.

## 2. Results and Discussion

### 2.1. Plastid Genome Structures of E. wushanense and Its Closely Related Species

**Figure 1:**
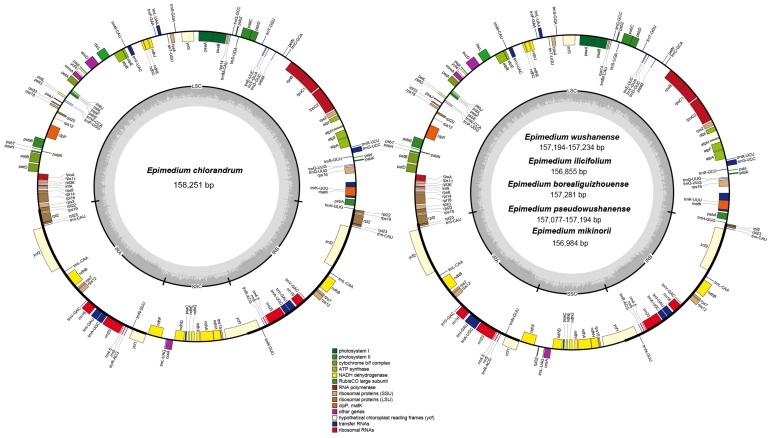
Gene map of the complete plastid genomes of *E. wushanense* and its closely related species. Genes drawn inside the circle are transcribed clockwise, whereas those outside are transcribed counterclockwise. The darker gray in the inner circle corresponds to the GC content, whereas the lighter gray corresponds to the AT content.

The plastid genome length of the six *Epimedium* species (*E. wushanense*, *E*. *pseudowushanense*, *E. chlorandrum*, *E. mikinorii*, *E. ilicifolium*, and *E. borealiguizhouense*) ranges from 156,855 bp to 158,251 bp. All the nine plastid genomes displayed a typical quadripartite structure, which consists of a pair of inverted repeats (IRs, 25,775–27,693 bp) separated by a large single-copy (LSC) region (86,627–88,603 bp) and a small single-copy (SSC) region (16,238–17,091 bp) (Figure 1, Table 1). The average GC content of the nine plastid genomes was 38.80%. Congruent with previous research, a total of 112 genes were identified from the nine plastid genomes, including 78 protein-coding, 30 tRNA, and 4 rRNA genes [18] (Figure 1). Similar with other angiosperms [19,20], a total of 18 genes containing introns were found. Among them, three genes, namely, *clpP*, *rps12*, and *ycf3*, contain two introns. The other 15 genes (*atpF*, *rpoC1*, *rpl2*, *rpl16*, *petB*, *petD*, *rps16*, *ndhA*, *ndhB*, *trnA-UGC*, *trnG-UCC*, *trnL-UAA*, *trnK-UUU*, *trnI-GAU*, and *trnV-UAC*) contain one intron. The loss of plastid *rpl32* gene in *Populus* and *Thalictrum* species has been found, and the corresponding *rpl32* gene has been transferred in the nuclear genome [21,22,23]. *E. chlorandrum* was treated as a synonym of *E. acuminatum* in the research of Zhang et al. [24]. Compared with *E. acuminatum*, an 859 bp deletion (including *ndhF-rpl32* intergenic spacer, partial sequence; *rpl32* gene, complete sequence; and *rpl32-trnL* intergenic spacer, partial sequence) in *E. chlorandrum* was found for the first time in our study. The deletion was verified through PCR amplification and Sanger sequencing with specific primers rpl32_1F/rpl32_1R (Table S1). The special plastid genome structure of *E. chlorandrum* suggests that it is different from *E. acuminatum*.

**Figure 2:**
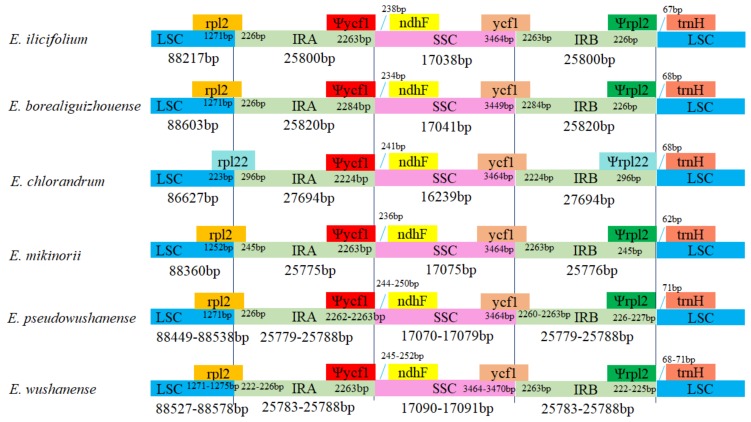
Comparison of the borders of the LSC, SSC and IR regions among plastid genomes of *E. wushanense* and its closely related species.

The contraction and expansion at the boundary of the IR regions were considered the main reasons for the length variation of the plastid genomes [25]. In this study, the gene *ycf1* crossed the SSC/IR region, and the pseudogene fragment *ycf1* was located at the IRA region with 2224–2284 bp in the plastid genomes of the six *Epimedium* species. Meanwhile, evident variations at the IR and LSC boundary regions were observed among the plastid genomes of *E. chlorandrum* and the other five species (*E. wushanense*, *E. pseudowushanense*, *E. borealiguizhouense*, *E. ilicifolium*, and *E. mikinorii*). The gene *rpl22* crossed the LSC/IRA region, and *Ψrpl22* with 296 bp was located at the IRB region in the plastid genome of *E. chlorandrum*, which was congruent with *E. acuminatum*. Otherwise, the gene *rpl2* crossed the LSC/IRA region in the other five species, and *Ψrpl2* with 222–245 bp was located in the IRB region. This finding is congruent with the *E. dolichostemon*, *E. lishischenii*, and *E. pseudowushanense* investigated by Zhang et al. [18]. At the junction of the IRA/SSC region, the distance between *ψycf1* and *ndhF* ranged from 234 to 252b p. In addition, the distance between *ψrpl22* and *trnH* in *E. chlorandrum* was 68 bp, whereas that between *ψrpl2* and *trnH* in the other four species ranged from 62 to 71 bp at the junction of the IRB/LSC region (Figure 2).

### 2.2. Codon Usage

**Figure 3:**
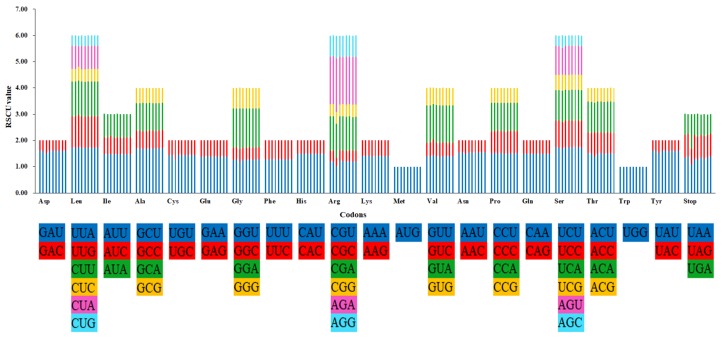
Codon content of 20 amino acids and stop codons in all protein-coding genes of the plastid genomes of *E. wushanense* and its closely related species. The histogram from the left to right side represents plastid genomes of *E. wushanense* (HBXS, HBXS-2), *E. borealiguizhouense*, *E. chlorandrum*, *E. mikinorii* (HBES, GZLS), *E*. *pseudowushanense* (GZJH, GZNW) and *E. ilicifolium*, respectively.

All the protein-coding genes consist of 26,058 (*E. borealiguizhouense*) to 26,333 (*E. chlorandrum*) codons in the nine *Epimedium* plastid genomes. These codons encode 20 amino acids (Table S2, Figure 3). The most and least amino acids in *Epimedium* species are respectively leucine (9.3–10.5%) and cysteine (1.1–1.5%). Apart from methionine and tryptophan, other amino acid codons all have preferences. The results showed a strong bias in codon usage for A and U at the third codon position (Figure 3). Similar results for codon usage were also observed in the plastid genomes of *Taxillus* and *Panax* species [19,25]. Interestingly, the usage of codons in *E. borealiguizhouense* showed preference for higher G/C content at the 3rd codon position, as well as AGA and stop codon UGA, comparing with the other five species (Figure 3).

### 2.3. SSRs and Repeat Structure Analyses

SSRs, consisting of tandem repeated DNA sequences in the plastid genome, present high levels of polymorphism [26]. SSRs have been used as molecular markers for species identification and phylogenetic investigations [26,27]. In this study, 71–79 SSRs were detected in the nine *Epimedium* plastid genomes using the MISA tool (Table S3). The majority of SSRs in all species were mononucleotides repeats (88.1–92.4%), and the A/T mononucleotides repeats were the most common, with a proportion of 84.6–89.9% (Table 2). Pentanucleotide SSRs were detected in all the species sequenced in our study except for *E. borealiguizhouense*. Aside from population GZNW of *E. pseudowushanense*, no hexanucleotide SSR existed in the other five species. Congruent with previous research [18], most of these SSR loci were located in the LSC region, followed by the SSC and IR regions.

**Figure 4:**
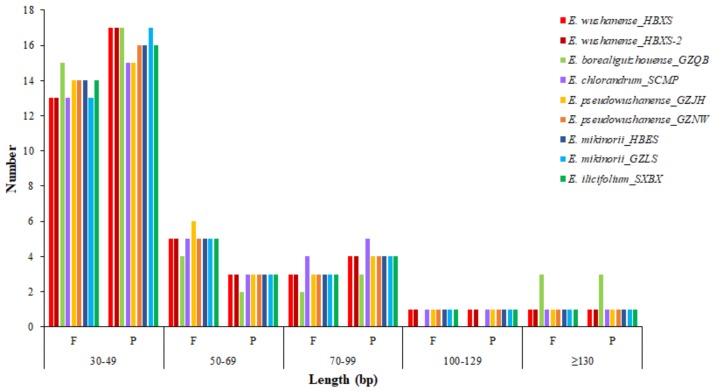
Repeat sequences in the plastid genomes of *E. wushanense* and its closely related species. F, forward; P, palindrome.

Repetitive structures in plastid genomes is closely related to the plastid genome rearrangement and variation, which is of great importance for phylogenetic and population genetics studies [28]. A total of 49 repeats were identified in each of the nine *Epimedium* plastid genomes by REPuter (Table S4). *E. chlorandrum*, *E. borealiguizhouense*, and *E. ilicifolium* all possess 24 forward and 25 palindromic repeats. Both of the two populations of *E. wushanense* consist of 23 forward and 26 palindromic repeats; whereas the two populations of *E. pseudowushanense* and *E. mikinorii* comprise different number of forward and palindromic repeats (Figure 4). The lengths of repeats in the plastid genome of *E. borealiguizhouense* ranged from 32 bp to 189 bp, whereas those in the other eight plastid genomes ranged from 32 bp to 131 bp (Figure 4, Table S4). The special repetitive structure of *E. borealiguizhouense* could distinguish it from *E. wushanense*. The copy lengths with 30–49 bp are the most common (57.1–65.3%), whereas those with lengths longer than 100 bp are minimal (8.2–12.2%) (Figure 4). These repeats were located in the LSC and IR regions, and no repeat was found in the SSC region. Interestingly, the intergenic spacer (IGS) and protein coding (pCD) regions are the most common distribution places for repeats. Previous studies showed that repeated sequences in many angiosperm lineages are mainly distributed in the IGS and intron regions of plastid genomes [19,20,29]. However, a larger number of repeats were found in the pCD regions than those in the noncoding regions in the present study, which is similar with the report of Zhang et al. [18] (Table S4).

### 2.4. Genome Sequence Divergence

**Figure 5:**
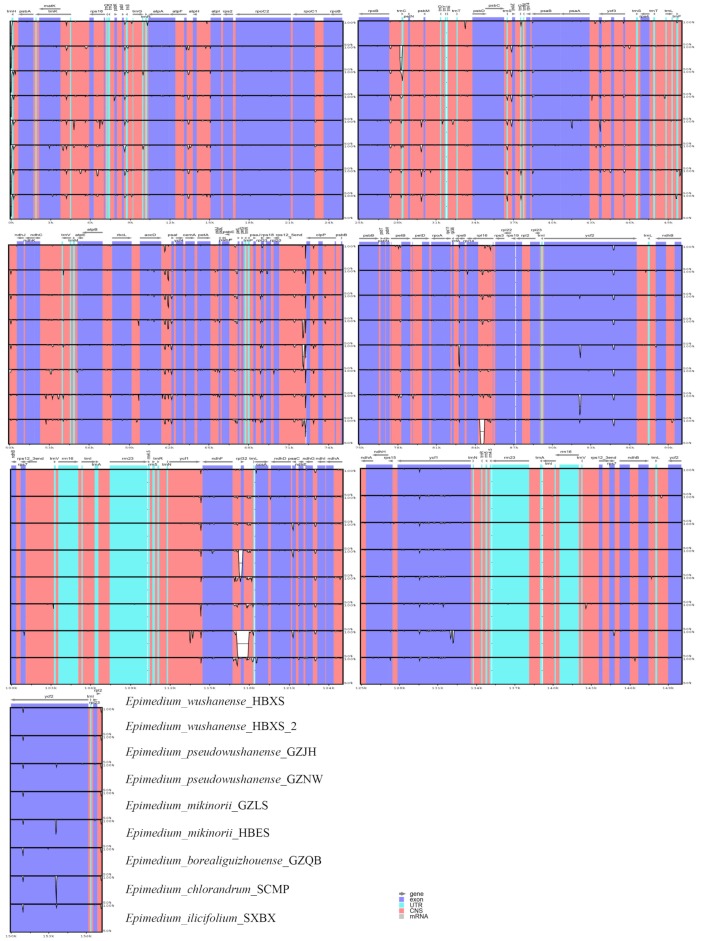
Sequence identity plots comparing nine *Epimedium* plastid genomes with *E. wushanense* as a reference.

**Figure 6:**
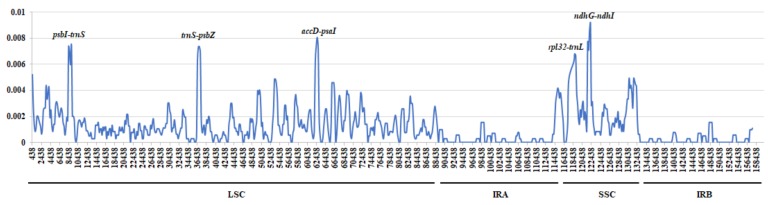
Nucleotide diversity graphs of nine *Epimedium* plastid genomes. The *x*-axis represents position of the midpoint of a window, and the *y*-axis represents Pi value.

The nine *Epimedium* plastid genomes obtained from the present study were compared and plotted using mVISTA with *E. wushanense* (HXBX) as a reference (Figure 5). The plots illustrate the high similarity of the nine *Epimedium* plastid genome sequences. The sequence similarity ranged from 99.4% (*E. chlorandrum*) to 99.8% (*E. wushanense*, HXBX-2) among these samples. Meanwhile, the coding region exhibited a lower divergence than the non-coding region, and sequence similarity was close to 100% in the coding region. The highly variable regions in the nine *Epimedium* plastid genomes were identified by the sliding window analysis using DnaSP. The average Pi value for nine *Epimedium* plastid genomes was 0.00121. The IR regions exhibited lower nucleotide diversity than the LSC and SSC regions. Meanwhile, the results showed that the most divergent regions were localized in the IGS region. A total of five regions showed higher Pi values than other regions (Pi > 0.006), namely, *psbI-trnS*, *trnS-psbZ*, *accD-psaI*, *rpl32-trnL*, and *ndhG-ndhI* (Figure 6). These divergent regions, which comprise abundant variation information, can be used to develop molecular markers for authenticating *E. wushanense* and its closely related species.

### 2.5. Phylogenetic and Taxonomic Implications for the Genus Epimedium

**Figure 7:**
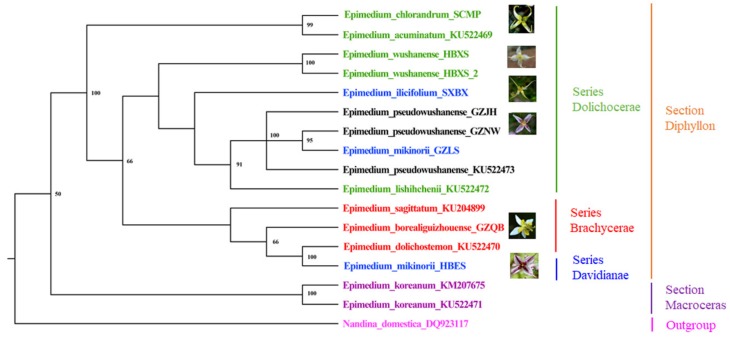
Phylogenetic relationships of 11 *Epimedium* species constructed based on the 87 protein-coding genes with maximum likelihood (ML). Numbers at the nodes represent ML bootstrap values. Stearn’s classification for each species is represented by different color, and species in the same series are represented by the same color.

The classification and identification of the genus *Epimedium* has always been controversial. The plastid genomes contained much variation, which can be used to discriminate genetically close species [30]. In this study, the results showed that *E. wushanense* can be distinguished from its relative species (Figure 7). Phylogenetic analysis based on karyomorphology [31] and molecular markers [4,32] has been done to confirm the evolution of this genus. However, the further subdivision of the Chinese sect. *Diphyllon* was poorly resolved. In this study, protein-coding genes of 17 plastid genomes from 11 *Epimedium* species and one outgroup species (*Nandina domestica*) were used to construct the phylogenetic trees using maximum likelihood (ML) method. The phylogenetic tree generated by the ML, including ML bootstrap values, is shown in Figure 7. Within *Epimedium*, the 11 species were divided into four clades. *E. koreanum* was first separated from the other *Epimedium* species, which was congruent with Stearn’s classification system. However, the relationship of the other species belonging to sect. *Diphyllon* did not completely support Stearn’s classification. *E. wushanense*, *E. lishihchenii*, *E. chlorandrum*, and *E. acuminatum* were divided into ser. *Dolichocerae* by Stearn [1]. Three specimens of *E. pseudowushanense* and two specimens of *E. wushanense* clustered as single branch respectively, supporting *E. pseudowushanens* as a new species in-dependent of *E. wushanense* [6]. Based upon the floral characters, *E. pseudowushanense* was classified into ser. *Dolichocerae* by Guo et al. [6]. Zhang et al. [10] proposed that *E. pseudowushanense* should be grouped into ser. *Davidianae* and that *E. wushanense* should be moved from ser. *Dolichocerae* to ser. *Davidianae*. In our study, *E. pseudowushanense* was clustered with *E. lishihchenii* and *E. wushanense* that were grouped into ser. *Dolichocerae*, supporting the taxonomic views of Stearn [1] and Guo et al. [6]. Moreover, *E. ilicifolium* was clustered with species belonging to ser. *Dolichocerae* into one branch, which supports the opinion of moving it from ser. *Davidianae* to ser. *Dolichocerae* [10]. In previous research, Guo et al. [3] grouped *E. mikinorii* from ser. *Davidianae* [1] to ser. *Dolichocerae* according to its petal characteristics. However, *E. mikinorii* was first clustered with *E. dolichostemon* in one branch and then clustered with *E. borealiguizhouense* in one clade. Hence, *E. mikinorii* was inferred to have a close relationship with species belonging to ser. *Brachycerae*. The population GZLS, a transitional species between *E. pseudowushanense* and *E. mikinorii*, was clustered with *E. pseudowushanense* into one branch, demonstrating that it may have a closer evolutionary relationship to *E. pseudowushanense*. The present study provides valuable information for identifying *Epimedium* species and resolving their phylogenetic relationships. To further explore the phylogeny of the whole genus, more plastid genomes of *Epimedium* species are needed. At present, the plastid genomes of plants have been applied as a useful tool for exploring the phylogenetic relationships in many genera [15,16,17,30]. Some studies report the conflicts in phylogenetic trees based on plastid and nuclear genome data [33,34]. In the future work, we will thoroughly analyze the phylogenetic relationships of the genus *Epimedium* derived from both plastid and nuclear genome data, thus comprehensively understanding the evolutionary relationship of this genus.

## 3. Materials and Methods

### 3.1. Taxon Sampling, DNA Extraction, and Sequencing

Totally 16 specimens representing 11 *Epimedium* species were collected. Among them, nine samples from six *Epimedium* species were sampled from Guizhou, Hubei, Shaanxi, and Sichuan provinces of China (Table S5). These samples were all identified by taxonomist Prof. Yanqin Xu at Jiangxi University of Traditional Chinese Medicine and taxonomist Prof. Shunzhi He at Guiyang College of Traditional Chinese Medicine. All corresponding voucher samples were deposited in the Herbarium of the Institute of Medicinal Plant Development, Chinese Academy of Medicinal Sciences, Beijing, China. Before DNA extraction, the fresh leaves were frozen at −20 °C Total genomic DNA was extracted and purified using the Plant Genomic DNA Rapid Extraction kit (Bioteke Corporation, Beijing, China) in accordance with the manufacturer’s instructions. The quantified DNA was then used to construct shotgun libraries with an average insert size of 500 bp and sequenced using Illumina Hiseq X in accordance with the manufacturer’s manual. Approximately 5 GB of raw data from each sample were produced with 150 bp pair-end read lengths. Moreover, seven plastid genomes from six *Epimedium* species [18,35,36] were downloaded from GenBank (Table S5).

### 3.2. Genome Assembly and Genome Annotation

Low-quality reads including a mixture data of nuclear and organelle genomes were trimmed with the software Trimmomatic [37]. The clean reads were then extracted and mapped to the reference database. Such database is constructed with all plastid genomes of plants from the National Center for Biotechnology Information on the basis of their coverage and similarity. The extracted reads were assembled to contigs with SOAPdenovo (v2, BGI HK Research Institute, Hong Kong, China) [38]. Complete plastid genome sequences were obtained with the combination and extension of the resulting contigs. Gaps in the assemblies were bridged by Sanger sequencing with specific primers designed for PCR based on their flanking sequences. To ensure assembly accuracy, the four junctions between the inverted repeats (IRs) and single-copy (SC) regions were verified through PCR amplification and Sanger sequencing with specific primers. All the specific primers are listed in Table S1.

The plastid genome annotation was performed using the software CPGAVAS [39], with manual corrections using the Apollo genome editor [40]. The plastid genome maps were generated using the Organellar-Genome DRAW (v1.2, Max Planck Institute of Molecular Plant Physiology, Potsdam, Germany) [41] with default settings and checked manually. The complete and correct plastid genome sequences of the six species were deposited in GenBank under accession numbers of MK408750-MK408754 and MK992918-MK992921 (Table S5). Raw sequences are available in the Sequence Read Archive of the NCBI under the accession numbers SAMN12430115-SAMN12430123 (Table S5).

### 3.3. Genome Structure Analyses and Genome Comparison

The GC content was analyzed using the software Mega 6.0 [42]. Simple sequence repeats (SSRs) were identified using the MISA Perl script (http://pgrc.ipk-gatersleben.de/misa/). Microsatellites were detected, including mono-, di-, tri-, tetra-, penta-, and hexanucleotide repeats, with thresholds of 10 repeat units for mono-, six repeat units for di-, four repeat units for tri- and tetra-, and three repeat units for penta- and hexanucleotide SSRs. The REPuter program [43] was run to identify the location and size of forward, palindromic, reverse, and complement repeats in the plastid genomes under the following criterion: Cutoff *n* ≥ 30 bp and 90% sequence identities (Hamming distance of 3). The alignment for the plastid genomes was performed and plotted using the mVISTA program [44]. The distribution of codon usage for all protein-coding genes was investigated using MEGA 6.0 [42] with the relative synonymous codon usage (RSCU) ratio [45]. To calculate the nucleotide diversity (Pi) and detect highly variable sites among *Epimedium* plastid genomes, DNA polymorphism analysis was performed using DnaSP (DNA Sequence Polymorphism) v6 [46] with 200 bp step size and 800 bp window length.

### 3.4. Phylogenetic Analysis

The 87 protein-coding genes were extracted from plastid genomes generated from the present research and downloaded from GenBank. The sequences were concatenated and aligned using MAFFT v7 [47], and *Nandina domestica* (DQ923117) was used as an outgroup. Appropriate nucleotide substitution models and parameters for the ML was conducted by running Likelihood Ratio Tests in PAUP^*^4.0 b10 [48] and using MrModeltest [49]. Model testing showed that the general time reversible (GTR) including rate variation among sites (+G) and invariable sites (+I) (= GTR + G + I) was the best fit model to the data sets under the Akaike Information Criterion (AIC). The ML analysis was conducted using the best-fitting model with 1000 bootstrap replicates.

## 4. Conclusions

This work reports nine new complete plastid genomes from *E. wushanense* and its closely related species, thereby enriching the plastid genome data of the *Epimedium* genus. A loss of *rpl32* gene in *E. chlorandrum*, which is a unique feature to distinguish it from other species, was found. The phylogenetic tree constructed demonstrated that *E. wushanense* can be separated from its relative species. Moreover, the plastid genome data provide new evidence for classifying *Epimedium* species, which will contribute valuable information for further exploring the phylogenetic relationships of the genus *Epimedium*.

## Figures and Tables

**Table 1:** Plastid genome characteristics of *E. wushanense* and its closely related species.

Latin Name	Voucher No.	Accession No.	LSC Length/bp	SSC Length/bp	IR Length/bp	Genome Size/bp	GC Content/%
*E. wushanense*	HBXS	MK408753	88,527	17,091	25,788	157,194	38.80
*E. wushanense*	HBXS-2	MK992920	88578	17,090	25,783	157,234	38.80
*E. pseudowushanense*	GZJH	MK408750	88,449	17,070	25,779	157,077	38.78
*E. pseudowushanense*	GZNW	MK992919	88,538	17,079	25,788	157,193	38.78
*E. mikinorii*	HBES	MK408752	88,360	17,074	25,775	156,984	38.81
*E. mikinorii*	GZLS	MK992918	88,522	16,833	25,783	156,921	38.80
*E. chlorandrum*	SCMP	MK408754	86,627	16,238	27,693	158,251	38.89
*E. ilicifolium*	SXBX	MK992921	88,217	17,038	25,800	156,855	38.77
*E. borealiguizhouense*	GZQB	MK408751	88,603	17,040	25,819	157,281	38.78

**Table 2:** Types and amounts of SSRs in the *E. wushanense* and its closely related species.

SSR Type	Repeat Unit	Amount/Ratio (%)								
		*E. borealiguizhouense*	*E. mikinorii*		*E. chlorandrum*	*E. pseudowushanense*		*E. wushanense*		*E. ilicifolium*
		GZQB	HBES	GZLS	SCMP	GZJH	GZNW	HBXS	HBXS-2	SXBX
Mono	A/T	64/88.9	61/85.9	67/88.2	66/84.6	68/88.3	67/87.0	69/88.5	71/89.9	65/85.5
	C/G	2/2.8	2/2.8	2/2.6	3/3.8	2/2.6	2/2.6	2/2.6	2/2.5	2/2.6
Di	AT/AT	1/1.4	2/2.8	2/2.6	1/1.3	2/2.6	2/2.6	2/2.6	3/3.8	2/2.6
Tri	AAT/ATT	1/1.4	1/1.4	1/1.3	2/2.6	1/1.3	1/1.3	1/1.3	0/0	1/1.3
	AGG/CCT	3/4.2	3/4.2	3/3.9	3/3.8	3/3.9	3/3.9	3/3.8	3/3.8	3/3.9
	AAG/CTT	1/1.4	1/1.4	0/0	1/1.3	0/0	0/0	0/0	0/0	1/1.3
Tetra	AAAT/ATTT	0/0	0/0	0/0	1/1.3	0/0	0/0	0/0	0/0	1/1.3
Penta	AAGAT/ATCTT	0/0	1/1.4	1/1.3	1/1.3	1/1.3	1/1.3	1/1.3	0/0	0/0
	AATAT/ATATT	0/0	0/0	0/0	0/0	0/0	0/0	0/0	0/0	1/1.3
Hexa	AACGAC/CGTTGT	0/0	0/0	0/0	0/0	0/0	1/1.3	0/0	0/0	0/0
Total		72/100	71/100	76/100	78/100	77/100	77/100	78/100	79/100	76/100

## Supplementary Materials

Supplementary Materials can be found at https://www.mdpi.com/1422-0067/20/16/4003/s1. Table S1: Primers used for genome sequence validation, Table S2: Codon usage within the plastid genomes of *E. wushanense* and its closely related species, Table S3: Distribution of SSR loci in the plastid genomes of *E. wushanense* and its closely related species, Table S4: Repeated sequences identified in the plastid genomes of *E. wushanense* and its closely related species, Table S5: Voucher information and GenBank accession numbers for the *Epimedium* samples.
