# Supplementary material for: Development of Plastid Genomic Resources for Discrimination and Classification of Epimedium wushanense (Berberidaceae)
Source: Int J Mol Sci. 2019 Aug 16;20(16):4003. doi: 10.3390/ijms20164003 (PMC6720487; doi:10.3390/ijms20164003)
Supplement: Supplementary file 1 [file ijms-20-04003-s001.pdf]

International Journal of *Molecular Sciences*

MDPI

Article

Development of Plastid Genomic Resources for Discrimination and Classification of *Epimedium wushanense* (Berberidaceae)

Mengyue Guo [1,†], Li Ren [1,†], Yanqin Xu [2], Baosheng Liao [3], Jingyuan Song [1,4], Ying Li [1,4], Nitin Mantri [5], Baolin Guo [1], Shilin Chen [3,4] and Xiaohui Pang [1,4,*]

[1] Key Lab of Chinese Medicine Resources Conservation, State Administration of Traditional Chinese Medicine of the People's Republic of China, Institute of Medicinal Plant Development, Chinese Academy of Medical Sciences & Peking Union Medical College, Beijing 100193, China
[2] College of Pharmacy, Jiangxi University of Traditional Chinese Medicine, Nanchang 330004, China
[3] Institute of Chinese Materia Medica, China Academy of Chinese Medical Sciences, Beijing 100700, China
[4] Engineering Research Center of Tradition Chinese Medicine Resource, Ministry of Education, Institute of Medicinal Plant Development, Chinese Academy of Medical Sciences & Peking Union Medical College, Beijing 100193, China
[5] The Pangenomics Group, School of Science, RMIT University, Melbourne 3083, Australia
* Correspondence: xhpang@implad.ac.cn; Tel.: +86-10-5783-3051
† These authors contributed equally to this work.

Table S1. Primers used for genome sequence validation.

Name of primers	Primer sequences 5′-3′	Samples	Location
SMG1F	TGCATATTGATTTCCACCTA	SCMP	GAP
SMG1R	TCTTTAACACCCGCTTTG	SCMP	GAP
SMG2F	GGTATTCAATCGCATCAA	SCMP	GAP
SMG2R	GTTTGTAACAATAGGGAC	SCMP	GAP
rpl32_1F	TTGTTTCCGATTCACCAG	SCMP	Gene loss
rpl32_1R	TTCTAATCCCTATCCCAC	SCMP	Gene loss
AH2F	CAGGCTTATGATATTTGTTC	GZJH, HBES, HBXS, SCMP, GZQB, HBXS-2, GZLS, SXBX	IRA-SSC
AH2R	GCGGTCGAATTTCTTCTT	GZJH, HBES, HBXS, SCMP, GZQB, HBXS-2, GZLS	IRA-SSC
BJ3F	ACGGAGACTGGTGCGAATG	GZNW, SXBX	IRA-SSC

BJ3R	TCCCAACCCGAGTATGAA	GZNW	IRA-SSC
DW3R	TAAGCCAAGTCCCAACCC	SXBX	IRA-SSC
GJ1-4F	AAATAAAGCGTTGGGTCG	GZJH, GZNW, GZLS	IRB-LSC
GJ1-4R	GGTTGGGTCGTTCTTGTG	GZJH, GZLS	IRB-LSC
GZQB-4F	CTGTTCGGTTATTGAGGA	GZQB, HBXS-2	IRB-LSC
GZQB-4R	AAGAGGTTGGGTCGTTCT	GZQB	IRB-LSC
HE4F	CGGGAAAGAGTCGAAGAA	HBES	IRB-LSC
HE4R	TTGTAAAGAGGTTGGGTC	HBES, HBXS-2	IRB-LSC
HX1-4F	AAAACTGTTCGGTTATTGAG	HBXS	IRB-LSC
HX1-4R	GAGGTTGGGTCGTTCTTG	HBXS, SCMP	IRB-LSC
SM4F	ATGATCGGCCATACAATT	SCMP	IRB-LSC
NW2R	TTGGGTTAAGAAGTTGAGAA	GZNW	IRB-LSC
DW2F	ACTTCATAACACGGCGATAC	SXBX	IRB-LSC
DW2R	CAAACTCACATAGGCATT	SXBX	IRB-LSC
GJ1-1F	CGGTTCTGTCGGTGCTTC	GZJH	LSC-IRA
GJ2-1R	ACAGAAATAAAGCGTTGG	GZQB, GZLS	LSC-IRA
GZQB-1F	ATTCGCCTCTGACCATCA	GZQB	LSC-IRA
HE1F	CTTTCAATTCGCCTCTGA	HBES	LSC-IRA
HE1R	GAAATAAAGCGTTGGGTC	HBES	LSC-IRA
HX1-1F	CGGTTCTATCGGTGCTTC	HBXS, HBXS-2	LSC-IRA
HX1-1R	ACTGTTCGGTTATTGAGG	HBXS, GZJH, HBXS-2	LSC-IRA
SM1F	CTATACGTCCAATTCCCTC	SCMP	LSC-IRA
SM1R	AATGATCGGCCATACAAT	SCMP	LSC-IRA
BJ1F	GAAATGTGAATAACTCGTCCTC	GZNW, GZLS, SXBX	LSC-IRA
BJ1R	CAGCGTCGTTGTGGTAAA	GZNW, SXBX	LSC-IRA
AH3R	CAGGCTTATGATATTTGTTC	GZJH, HBES, HBXS, SCMP, HBXS-2, GZNW, GZLS, SXBX	SSC-IRB
ZM1-3F	TCTTTACTCCACGCTTTC	GZJH, HBES, HBXS, SCMP, HBXS-2, GZLS	SSC-IRB
BJ4F	GTCTGCCACCTTCTTCTC	GZNW, SXBX	SSC-IRB

Table S2. Codon usage within the cp genomes of *E. wushanense* and its closely related species.

Amino acid	Con don	Voucher No.																	
		HBXS		HBXS-2		GZQB		SCMP		HBES		GZLS		GZJH		GZNW		SXBX	
		Num ber	RSCU value	Num ber	RSCU value	Num ber	RSCU value	Num ber	RSCU value	Num ber	RSCU value	Num ber	RSCU value	Num ber	RSCU value	Num ber	RSCU value	Num ber	RSCU value
Phe	UUU	981	1.28	988	1.28	1063	1.29	978	1.28	982	1.28	988	1.28	982	1.28	987	1.28	988	1.28
	UUC	551	0.72	553	0.72	582	0.71	551	0.72	553	0.72	554	0.72	552	0.72	554	0.72	553	0.72
Leu	UUA	791	1.73	787	1.72	703	1.74	795	1.72	790	1.72	790	1.72	792	1.73	788	1.72	787	1.72
	UUG	541	1.18	545	1.19	495	1.22	549	1.19	542	1.18	546	1.19	544	1.19	547	1.19	545	1.19
	CUU	610	1.33	612	1.33	536	1.32	612	1.33	612	1.33	612	1.33	610	1.33	611	1.33	612	1.33
	CUC	218	0.48	218	0.48	216	0.53	219	0.47	219	0.48	218	0.48	218	0.47	218	0.48	218	0.48
	CUA	406	0.89	405	0.88	318	0.78	410	0.89	405	0.88	403	0.88	407	0.89	406	0.88	406	0.89
	CUG	185	0.4	185	0.4	163	0.4	186	0.4	184	0.4	183	0.4	183	0.4	183	0.4	183	0.4
Ile	AUU	1074	1.48	1071	1.47	1003	1.47	1072	1.47	1070	1.48	1072	1.47	1073	1.48	1068	1.48	1075	1.48
	AUC	462	0.64	463	0.64	477	0.7	461	0.63	460	0.64	464	0.64	462	0.64	459	0.63	463	0.64
	AUA	644	0.89	646	0.89	563	0.83	653	0.9	642	0.89	645	0.89	642	0.88	644	0.89	643	0.88
Met	AUG	602	1	604	1	525	1	607	1	605	1	605	1	603	1	604	1	605	1
Val	GUU	485	1.39	482	1.4	462	1.45	483	1.39	480	1.38	482	1.4	484	1.39	478	1.39	482	1.39
	GUC	182	0.52	178	0.52	187	0.59	180	0.52	182	0.52	178	0.52	182	0.52	177	0.51	179	0.52
	GUA	495	1.42	492	1.42	427	1.34	498	1.44	496	1.43	492	1.42	495	1.42	492	1.43	492	1.42
	GUG	229	0.66	230	0.67	202	0.63	227	0.65	231	0.67	230	0.67	229	0.66	231	0.67	230	0.67
Ser	UCU	593	1.74	593	1.73	626	1.69	595	1.74	592	1.74	595	1.74	596	1.75	595	1.74	593	1.73
	UCC	345	1.01	350	1.02	380	1.02	346	1.01	345	1.01	349	1.02	344	1.01	349	1.02	351	1.02
	UCA	398	1.17	398	1.16	440	1.19	403	1.18	398	1.17	400	1.17	400	1.17	400	1.17	402	1.17
	UCG	197	0.58	199	0.58	226	0.61	196	0.57	197	0.58	199	0.58	197	0.58	197	0.58	197	0.57
	AGU	375	1.1	377	1.1	375	1.01	378	1.1	375	1.1	376	1.1	375	1.1	377	1.1	377	1.1
	AGC	135	0.4	137	0.4	179	0.48	135	0.39	135	0.4	136	0.4	134	0.39	136	0.4	137	0.4
Pro	CCU	433	1.51	433	1.52	389	1.49	439	1.52	428	1.5	431	1.51	431	1.51	431	1.51	433	1.52
	CCC	238	0.83	238	0.83	230	0.88	243	0.84	240	0.84	240	0.84	240	0.84	239	0.84	240	0.84
	CCA	314	1.1	312	1.09	277	1.06	309	1.07	310	1.09	310	1.09	311	1.09	309	1.09	309	1.08
	CCG	160	0.56	160	0.56	149	0.57	161	0.56	160	0.56	159	0.56	159	0.56	159	0.56	159	0.56
Thr	ACU	493	1.51	492	1.51	443	1.38	495	1.5	493	1.52	491	1.5	493	1.51	491	1.51	490	1.5
	ACC	258	0.79	256	0.78	298	0.93	262	0.8	257	0.79	257	0.79	257	0.79	256	0.79	257	0.79
	ACA	387	1.19	386	1.18	358	1.12	390	1.18	382	1.17	385	1.18	385	1.18	385	1.18	386	1.18
	ACG	168	0.51	171	0.52	184	0.57	171	0.52	169	0.52	172	0.53	169	0.52	172	0.53	174	0.53
Ala	GCU	594	1.69	593	1.69	563	1.67	597	1.68	594	1.69	593	1.69	595	1.69	593	1.69	593	1.7
	GCC	238	0.68	238	0.68	225	0.67	243	0.68	239	0.68	237	0.68	237	0.67	237	0.68	237	0.68

	GCA	368	1.05	370	1.05	367	1.09	377	1.06	371	1.06	370	1.05	370	1.05	370	1.05	368	1.05
	GCG	203	0.58	202	0.58	191	0.57	208	0.58	202	0.57	203	0.58	203	0.58	203	0.58	201	0.57
Tyr	UAU	732	1.6	729	1.59	782	1.53	733	1.6	731	1.6	729	1.59	730	1.6	729	1.59	735	1.6
	UAC	183	0.4	186	0.41	241	0.47	186	0.4	184	0.4	187	0.41	184	0.4	187	0.41	185	0.4
His	CAU	469	1.49	466	1.49	478	1.5	477	1.5	469	1.49	466	1.49	468	1.49	466	1.49	465	1.49
	CAC	160	0.51	159	0.51	158	0.5	160	0.5	159	0.51	159	0.51	160	0.51	159	0.51	159	0.51
Gln	CAA	705	1.49	702	1.49	661	1.5	707	1.5	704	1.49	703	1.49	704	1.49	701	1.49	700	1.49
	CAG	241	0.51	241	0.51	218	0.5	238	0.5	240	0.51	240	0.51	240	0.51	240	0.51	240	0.51
Asn	AAU	911	1.55	912	1.55	871	1.5	919	1.55	912	1.55	914	1.55	910	1.55	911	1.55	910	1.55
	AAC	261	0.45	263	0.45	288	0.5	267	0.45	261	0.45	264	0.45	262	0.45	264	0.45	264	0.45
Lys	AAA	1017	1.41	1011	1.41	979	1.42	1013	1.4	1015	1.4	1010	1.41	1017	1.41	1004	1.4	1013	1.4
	AAG	430	0.59	427	0.59	398	0.58	431	0.6	430	0.6	427	0.59	429	0.59	427	0.6	429	0.6
Asp	GAU	900	1.6	896	1.59	736	1.51	895	1.6	894	1.6	895	1.59	896	1.6	891	1.59	898	1.6
	GAC	223	0.4	229	0.41	237	0.49	225	0.4	222	0.4	229	0.41	223	0.4	229	0.41	228	0.4
Glu	GAA	985	1.38	983	1.38	898	1.4	983	1.38	984	1.39	986	1.38	989	1.38	984	1.39	984	1.38
	GAG	442	0.62	440	0.62	385	0.6	441	0.62	436	0.61	440	0.62	440	0.62	436	0.61	439	0.62
Cys	UGU	214	1.45	215	1.45	250	1.26	216	1.46	214	1.45	214	1.45	214	1.45	214	1.45	213	1.44
	UGC	82	0.55	81	0.55	147	0.74	80	0.54	82	0.55	82	0.55	82	0.55	82	0.55	82	0.56
Trp	UGG	459	1	461	1	446	1	457	1	456	1	461	1	458	1	457	1	463	1
Arg	CGU	321	1.21	318	1.2	290	1.01	328	1.22	321	1.21	318	1.2	321	1.21	318	1.2	317	1.2
	CGC	107	0.4	107	0.41	99	0.34	108	0.4	107	0.4	107	0.4	107	0.4	107	0.4	107	0.4
	CGA	345	1.3	343	1.3	372	1.29	347	1.29	345	1.3	343	1.3	345	1.3	342	1.29	344	1.3
	CGG	124	0.47	124	0.47	128	0.44	123	0.46	124	0.47	124	0.47	124	0.47	125	0.47	124	0.47
	AGA	479	1.81	480	1.82	588	2.04	490	1.82	478	1.8	481	1.82	480	1.81	482	1.82	481	1.82
	AGG	213	0.8	213	0.81	252	0.87	215	0.8	214	0.81	214	0.81	215	0.81	213	0.81	213	0.81
Gly	GGU	552	1.26	554	1.27	522	1.19	566	1.27	553	1.26	554	1.27	553	1.26	554	1.27	555	1.27
	GGC	205	0.47	205	0.47	217	0.49	205	0.46	205	0.47	205	0.47	205	0.47	205	0.47	204	0.47
	GGA	653	1.49	647	1.48	672	1.53	659	1.48	653	1.49	647	1.48	654	1.49	647	1.48	648	1.48
	GGG	343	0.78	342	0.78	350	0.8	352	0.79	344	0.78	340	0.78	340	0.78	340	0.78	341	0.78
Stop	UAA	37	1.34	41	1.4	207	1.08	36	1.3	35	1.27	40	1.36	36	1.3	40	1.36	41	1.4
	UAG	24	0.87	25	0.85	112	0.59	25	0.9	24	0.87	25	0.85	24	0.87	25	0.85	25	0.85
	UGA	22	0.8	22	0.75	254	1.33	22	0.8	24	0.87	23	0.78	23	0.83	23	0.78	22	0.75

Table S3. Distribution of SSR loci in the cp genomes of *E. wushanense* and its closely related species.

Genomes/SSR No.	SSR type	Repeat motif	size (bp)	Start	End	Region
Epimedium wushanense_HBXS						
1	p1	(A)11	11	251	261	LSC

2	p1	(A)10	10	4013	4022	LSC
3	p1	(A)12	12	4855	4866	LSC
4	p1	(T)10	10	5599	5608	LSC
5	p1	(T)12	12	6532	6543	LSC
6	p1	(T)10	10	7466	7475	LSC
7	p1	(A)10	10	8030	8039	LSC
8	p1	(A)12	12	8578	8589	LSC
9	p1	(T)10	10	8603	8612	LSC
10	p1	(A)11	11	8804	8814	LSC
11	p1	(A)10	10	13013	13022	LSC
12	p1	(A)10	10	13678	13687	LSC
13	p5	(AGATA)3	15	14773	14787	LSC
14	p1	(T)10	10	18845	18854	LSC
15	p1	(T)13	13	22984	22996	LSC
16	p1	(T)10	10	26635	26644	LSC
17	p1	(A)11	11	28199	28209	LSC
18	p1	(A)10	10	29142	29151	LSC
19	p1	(T)10	10	29560	29569	LSC
20	p1	(T)10	10	30261	30270	LSC
21	p1	(T)12	12	31687	31698	LSC
22	p1	(G)11	11	35486	35496	LSC
23	p1	(A)13	13	38508	38520	LSC
24	p1	(C)10	10	41478	41487	LSC
25	p1	(T)10	10	43122	43131	LSC
26	p3	(ATA)4	12	43345	43356	LSC
27	p1	(A)11	11	43721	43731	LSC
28	p1	(T)12	12	44052	44063	LSC
29	p1	(T)10	10	44528	44537	LSC
30	p1	(T)11	11	45549	45559	LSC
31	p1	(A)12	12	45618	45629	LSC
32	p1	(A)11	11	45875	45885	LSC
33	p1	(T)11	11	47629	47639	LSC

34	p1	(T)10	10	48341	48350	LSC
35	p2	(TA)6	12	49920	49931	LSC
36	p1	(T)11	11	50562	50572	LSC
37	p1	(T)10	10	54162	54171	LSC
38	p1	(A)12	12	57593	57604	LSC
39	p3	(GGA)4	12	60717	60728	LSC
40	p1	(A)12	12	61927	61938	LSC
41	p1	(A)11	11	62212	62222	LSC
42	p1	(T)10	10	63266	63275	LSC
43	p1	(A)10	10	65320	65329	LSC
44	p1	(T)10	10	65677	65686	LSC
45	p1	(A)10	10	66287	66296	LSC
46	p1	(A)12	12	66916	66927	LSC
47	p1	(T)13	13	66988	67000	LSC
48	p1	(A)11	11	68865	68875	LSC
49	p1	(T)14	14	69004	69017	LSC
50	p1	(T)12	12	72029	72040	LSC
51	p1	(A)11	11	72307	72317	LSC
52	p1	(T)14	14	73008	73021	LSC
53	p1	(T)10	10	73739	73748	LSC
54	p1	(T)11	11	73951	73961	LSC
55	p1	(T)10	10	77811	77820	LSC
56	p1	(A)10	10	78050	78059	LSC
57	p1	(T)12	12	78183	78194	LSC
58	p1	(A)10	10	82762	82771	LSC
59	p1	(T)14	14	83272	83285	LSC
60	p1	(A)13	13	84382	84394	LSC
61	p1	(T)15	15	85161	85175	LSC
62	p1	(T)11	11	85264	85274	LSC
63	p1	(T)10	10	85542	85551	LSC
64	p1	(A)10	10	92132	92141	IRA
65	p1	(A)13	13	112817	112829	IRA

66	p3	(AGG)4	12	114217	114228	IRA
67	p1	(T)14	14	116808	116821	SSC
68	p1	(A)10	10	116871	116880	SSC
69	p1	(A)11	11	116937	116947	SSC
70	p1	(T)11	11	117578	117588	SSC
71	p2	(AT)8	16	117798	117813	SSC
72	p1	(T)16	16	117923	117938	SSC
73	p1	(T)10	10	122334	122343	SSC
74	p1	(A)13	13	124770	124782	SSC
75	p1	(A)16	16	131316	131331	IRB
76	p3	(TCC)4	12	131492	131503	IRB
77	p1	(T)13	13	132892	132904	IRB
78	p1	(T)10	10	153580	153589	IRB
Epimedium_borealiguizhouense_GZQB						
1	p1	(A)10	10	4012	4021	LSC
2	p1	(A)12	12	4849	4860	LSC
3	p1	(T)10	10	7454	7463	LSC
4	p1	(A)10	10	8018	8027	LSC
5	p1	(A)12	12	8558	8569	LSC
6	p1	(T)11	11	8582	8592	LSC
7	p1	(A)10	10	8784	8793	LSC
8	p1	(T)10	10	10028	10037	LSC
9	p1	(A)11	11	12984	12994	LSC
10	p1	(A)10	10	13648	13657	LSC
11	p1	(A)14	14	15027	15040	LSC
12	p1	(T)10	10	18815	18824	LSC
13	p1	(T)10	10	22954	22963	LSC
14	p1	(T)10	10	26602	26611	LSC
15	p1	(A)11	11	28160	28170	LSC
16	p1	(T)10	10	30242	30251	LSC
17	p1	(T)12	12	31668	31679	LSC
18	p1	(G)11	11	35467	35477	LSC

19	p1	(A)11	11	38495	38505	LSC
20	p1	(C)10	10	41463	41472	LSC
21	p1	(T)11	11	43107	43117	LSC
22	p3	(ATA)6	18	43326	43343	LSC
23	p1	(A)13	13	43709	43721	LSC
24	p1	(T)11	11	45535	45545	LSC
25	p1	(A)14	14	45605	45618	LSC
26	p1	(A)10	10	45864	45873	LSC
27	p1	(T)10	10	47619	47628	LSC
28	p2	(TA)6	12	49907	49918	LSC
29	p1	(T)12	12	49995	50006	LSC
30	p1	(T)11	11	50560	50570	LSC
31	p1	(T)13	13	54035	54047	LSC
32	p1	(T)10	10	54168	54177	LSC
33	p1	(A)12	12	57595	57606	LSC
34	p3	(GGA)4	12	60720	60731	LSC
35	p1	(A)14	14	61923	61936	LSC
36	p3	(AAG)4	12	62204	62215	LSC
37	p1	(T)10	10	63268	63277	LSC
38	p1	(T)10	10	65473	65482	LSC
39	p1	(T)13	13	65679	65691	LSC
40	p1	(A)10	10	66292	66301	LSC
41	p1	(A)11	11	66921	66931	LSC
42	p1	(T)16	16	66991	67006	LSC
43	p1	(A)11	11	68300	68310	LSC
44	p1	(A)10	10	68872	68881	LSC
45	p1	(T)14	14	69016	69029	LSC
46	p1	(T)15	15	69910	69924	LSC
47	p1	(T)11	11	72053	72063	LSC
48	p1	(A)13	13	72375	72387	LSC
49	p1	(A)14	14	72922	72935	LSC
50	p1	(T)14	14	73086	73099	LSC

51	p1	(T)11	11	73817	73827	LSC
52	p1	(T)11	11	74030	74040	LSC
53	p1	(T)11	11	77890	77900	LSC
54	p1	(A)10	10	78130	78139	LSC
55	p1	(T)10	10	78263	78272	LSC
56	p1	(A)11	11	82840	82850	LSC
57	p1	(T)14	14	83351	83364	LSC
58	p1	(A)12	12	84462	84473	LSC
59	p1	(T)12	12	85089	85100	LSC
60	p1	(T)15	15	85242	85256	LSC
61	p1	(T)11	11	85345	85355	LSC
62	p1	(A)10	10	92212	92221	LSC
63	p1	(A)13	13	112903	112915	IRA
64	p3	(AGG)4	12	114303	114314	IRA
65	p1	(T)15	15	116904	116918	SSC
66	p1	(T)11	11	117665	117675	SSC
67	p1	(T)15	15	118004	118018	SSC
68	p1	(A)13	13	124848	124860	SSC
69	p1	(A)16	16	131387	131402	IRB
70	p3	(TCC)4	12	131569	131580	IRB
71	p1	(T)13	13	132969	132981	IRB
72	p1	(T)10	10	153663	153672	IRB
Epimedium chlorandrum_SCMP						
1	p1	(A)10	10	256	265	LSC
2	p1	(A)12	12	4018	4029	LSC
3	p1	(A)10	10	4855	4864	LSC
4	p1	(T)10	10	5604	5613	LSC
5	p1	(T)11	11	6534	6544	LSC
6	p1	(T)11	11	7467	7477	LSC
7	p1	(A)11	11	8032	8042	LSC
8	p1	(A)10	10	8491	8500	LSC
9	p1	(A)10	10	8581	8590	LSC

10	p1	(T)10	10	8602	8611	LSC
11	p1	(A)12	12	8803	8814	LSC
12	p1	(T)11	11	10058	10068	LSC
13	p1	(A)12	12	13015	13026	LSC
14	p1	(A)10	10	13675	13684	LSC
15	p5	(AGATA)3	15	14770	14784	LSC
16	p1	(A)10	10	15059	15068	LSC
17	p1	(T)10	10	18843	18852	LSC
18	p1	(T)10	10	22982	22991	LSC
19	p1	(T)10	10	26630	26639	LSC
20	p1	(T)10	10	30256	30265	LSC
21	p1	(T)11	11	31682	31692	LSC
22	p1	(G)11	11	35479	35489	LSC
23	p1	(A)13	13	38511	38523	LSC
24	p1	(C)10	10	41481	41490	LSC
25	p1	(T)11	11	43125	43135	LSC
26	p3	(ATA)4	12	43349	43360	LSC
27	p1	(A)12	12	43726	43737	LSC
28	p1	(T)11	11	45551	45561	LSC
29	p1	(A)13	13	45621	45633	LSC
30	p1	(A)11	11	45879	45889	LSC
31	p1	(T)10	10	47634	47643	LSC
32	p2	(TA)6	12	49922	49933	LSC
33	p1	(T)11	11	50582	50592	LSC
34	p4	(TAAA)4	16	52743	52758	LSC
35	p1	(T)13	13	54049	54061	LSC
36	p1	(T)10	10	54182	54191	LSC
37	p1	(A)14	14	57614	57627	LSC
38	p1	(G)10	10	57628	57637	LSC
39	p3	(GGA)4	12	60754	60765	LSC
40	p1	(A)14	14	61964	61977	LSC
41	p3	(AAG)4	12	62239	62250	LSC

42	p1	(T)10	10	63303	63312	LSC
43	p1	(A)10	10	65357	65366	LSC
44	p1	(T)11	11	65714	65724	LSC
45	p1	(A)10	10	66325	66334	LSC
46	p1	(A)12	12	66954	66965	LSC
47	p1	(T)16	16	67025	67040	LSC
48	p1	(A)13	13	68334	68346	LSC
49	p1	(A)12	12	68908	68919	LSC
50	p1	(T)15	15	69054	69068	LSC
51	p1	(A)10	10	71532	71541	LSC
52	p1	(T)11	11	72086	72096	LSC
53	p1	(A)14	14	72408	72421	LSC
54	p1	(A)10	10	72956	72965	LSC
55	p1	(T)14	14	73116	73129	LSC
56	p1	(T)11	11	73847	73857	LSC
57	p1	(T)10	10	74060	74069	LSC
58	p1	(T)10	10	77919	77928	LSC
59	p1	(A)10	10	78158	78167	LSC
60	p1	(T)10	10	78291	78300	LSC
61	p1	(A)11	11	82862	82872	LSC
62	p1	(T)14	14	83373	83386	LSC
63	p1	(A)12	12	84483	84494	LSC
64	p1	(T)12	12	85116	85127	LSC
65	p1	(T)10	10	85366	85375	LSC
66	p1	(T)10	10	85643	85652	LSC
67	p1	(A)13	13	112861	112873	IRA
68	p3	(AGG)4	12	114222	114233	IRA
69	p1	(T)15	15	116809	116823	SSC
70	p1	(A)10	10	116945	116954	SSC
71	p1	(T)12	12	117076	117087	SSC
72	p3	(TTA)5	15	117429	117443	SSC
73	p1	(T)10	10	117461	117470	SSC

74	p1	(A)14	14	123927	123940	SSC
75	p1	(T)10	10	126711	126720	SSC
76	p1	(A)13	13	130468	130480	IRB
77	p3	(TCC)4	12	130644	130655	IRB
78	p1	(T)13	13	132005	132017	IRB
Epimedium pseudowushanense_GZJH						
1	p1	(A)11	11	259	269	LSC
2	p1	(A)11	11	4021	4031	LSC
3	p1	(A)12	12	4861	4872	LSC
4	p1	(T)10	10	5605	5614	LSC
5	p1	(T)11	11	6541	6551	LSC
6	p1	(T)10	10	7474	7483	LSC
7	p1	(A)10	10	8038	8047	LSC
8	p1	(A)11	11	8586	8596	LSC
9	p1	(T)12	12	8614	8625	LSC
10	p1	(A)15	15	8817	8831	LSC
11	p1	(A)10	10	13030	13039	LSC
12	p1	(A)10	10	13696	13705	LSC
13	p5	(AGATA)3	15	14796	14810	LSC
14	p1	(A)13	13	15085	15097	LSC
15	p1	(T)10	10	18872	18881	LSC
16	p1	(T)11	11	23011	23021	LSC
17	p1	(T)10	10	26660	26669	LSC
18	p1	(A)10	10	28218	28227	LSC
19	p1	(T)10	10	29459	29468	LSC
20	p1	(T)10	10	30160	30169	LSC
21	p1	(T)12	12	31587	31598	LSC
22	p1	(G)11	11	35385	35395	LSC
23	p1	(A)15	15	38399	38413	LSC
24	p1	(C)10	10	41371	41380	LSC
25	p1	(T)10	10	43015	43024	LSC
26	p3	(ATA)4	12	43238	43249	LSC

27	p1	(T)10	10	43942	43951	LSC
28	p1	(T)11	11	45436	45446	LSC
29	p1	(A)14	14	45505	45518	LSC
30	p1	(A)11	11	45764	45774	LSC
31	p1	(T)10	10	47525	47534	LSC
32	p2	(TA)6	12	49813	49824	LSC
33	p1	(T)10	10	50448	50457	LSC
34	p1	(A)10	10	53120	53129	LSC
35	p1	(T)13	13	53914	53926	LSC
36	p1	(T)10	10	54057	54066	LSC
37	p1	(A)12	12	57488	57499	LSC
38	p3	(GGA)4	12	60611	60622	LSC
39	p1	(A)19	19	61782	61800	LSC
40	p1	(T)12	12	63126	63137	LSC
41	p1	(A)10	10	65182	65191	LSC
42	p1	(T)12	12	65334	65345	LSC
43	p1	(T)12	12	65542	65553	LSC
44	p1	(A)10	10	66154	66163	LSC
45	p1	(A)12	12	66783	66794	LSC
46	p1	(T)15	15	66854	66868	LSC
47	p1	(A)11	11	68725	68735	LSC
48	p1	(T)17	17	68870	68886	LSC
49	p1	(T)11	11	71895	71905	LSC
50	p1	(A)13	13	72212	72224	LSC
51	p1	(A)13	13	72759	72771	LSC
52	p1	(T)14	14	72922	72935	LSC
53	p1	(T)11	11	73659	73669	LSC
54	p1	(T)11	11	73872	73882	LSC
55	p1	(T)11	11	77732	77742	LSC
56	p1	(A)10	10	77972	77981	LSC
57	p1	(T)13	13	78105	78117	LSC
58	p1	(T)10	10	82648	82657	LSC

59	p1	(A)10	10	82689	82698	LSC
60	p1	(T)15	15	83199	83213	LSC
61	p1	(A)13	13	84317	84329	LSC
62	p1	(T)12	12	84937	84948	LSC
63	p1	(T)13	13	85090	85102	LSC
64	p1	(T)11	11	85191	85201	LSC
65	p1	(A)10	10	92058	92067	IRA
66	p1	(A)13	13	112730	112742	IRA
67	p3	(AGG)4	12	114130	114141	IRA
68	p1	(T)14	14	116720	116733	SSC
69	p1	(A)10	10	116848	116857	SSC
70	p1	(T)11	11	117487	117497	SSC
71	p2	(AT)7	14	117707	117720	SSC
72	p1	(T)15	15	117830	117844	SSC
73	p1	(A)13	13	124669	124681	SSC
74	p1	(A)13	13	131208	131220	IRB
75	p3	(TCC)4	12	131384	131395	IRB
76	p1	(T)13	13	132784	132796	IRB
77	p1	(T)10	10	153459	153468	IRB
Epimedium mikinorii_HBES						
1	p1	(A)10	10	4011	4020	LSC
2	p1	(A)11	11	4866	4876	LSC
3	p1	(T)10	10	7480	7489	LSC
4	p1	(A)11	11	8044	8054	LSC
5	p1	(A)12	12	8588	8599	LSC
6	p1	(T)11	11	8612	8622	LSC
7	p1	(A)14	14	8814	8827	LSC
8	p1	(A)10	10	13683	13692	LSC
9	p5	(AGATA)3	15	14778	14792	LSC
10	p1	(A)13	13	15066	15078	LSC
11	p1	(T)10	10	18853	18862	LSC
12	p1	(T)10	10	22992	23001	LSC

13	p1	(A)10	10	28198	28207	LSC
14	p1	(T)10	10	30267	30276	LSC
15	p1	(T)12	12	31685	31696	LSC
16	p1	(G)11	11	35475	35485	LSC
17	p1	(A)12	12	38492	38503	LSC
18	p1	(C)10	10	41461	41470	LSC
19	p1	(T)10	10	43090	43099	LSC
20	p3	(ATA)4	12	43313	43324	LSC
21	p1	(A)13	13	43669	43681	LSC
22	p1	(T)12	12	45495	45506	LSC
23	p1	(A)12	12	45565	45576	LSC
24	p1	(A)11	11	45822	45832	LSC
25	p1	(T)10	10	47577	47586	LSC
26	p2	(TA)6	12	49865	49876	LSC
27	p1	(T)10	10	50500	50509	LSC
28	p1	(A)11	11	53172	53182	LSC
29	p1	(T)10	10	53966	53975	LSC
30	p1	(A)12	12	57527	57538	LSC
31	p3	(GGA)4	12	60652	60663	LSC
32	p1	(A)15	15	61854	61868	LSC
33	p3	(CTT)4	12	62074	62085	LSC
34*	p1	(T)11	11	62086	62096	LSC
35	p1	(T)10	10	63191	63200	LSC
36	p1	(T)11	11	65601	65611	LSC
37	p1	(A)10	10	66212	66221	LSC
38	p1	(A)12	12	66841	66852	LSC
39	p1	(T)12	12	66912	66923	LSC
40	p1	(A)11	11	68787	68797	LSC
41	p1	(T)20	20	68932	68951	LSC
42	p1	(A)10	10	71416	71425	LSC
43	p1	(T)10	10	71970	71979	LSC
44	p1	(A)13	13	72222	72234	LSC

45	p1	(A)14	14	72769	72782	LSC
46	p1	(T)11	11	72933	72943	LSC
47	p1	(T)10	10	73661	73670	LSC
48	p1	(T)11	11	73873	73883	LSC
49	p1	(T)10	10	77733	77742	LSC
50	p1	(A)10	10	77972	77981	LSC
51	p1	(T)11	11	78105	78115	LSC
52	p1	(T)14	14	83129	83142	LSC
53	p1	(A)13	13	84239	84251	LSC
54	p1	(T)11	11	84867	84877	LSC
55	p1	(T)15	15	85019	85033	LSC
56	p1	(T)10	10	85122	85131	LSC
57	p1	(A)10	10	91988	91997	IRA
58	p1	(A)13	13	112637	112649	IRA
59	p3	(AGG)4	12	114037	114048	IRA
60	p1	(T)14	14	116619	116632	SSC
61	p1	(A)11	11	116754	116764	SSC
62	p1	(T)11	11	117386	117396	SSC
63	p2	(AT)6	12	117606	117617	SSC
64	p1	(T)13	13	117727	117739	SSC
65	p1	(T)10	10	122143	122152	SSC
66	p1	(A)13	13	124573	124585	SSC
67	p1	(T)11	11	127361	127371	SSC
68	p1	(A)13	13	131119	131131	IRB
69	p3	(TCC)4	12	131295	131306	IRB
70	p1	(T)13	13	132695	132707	IRB
71	p1	(T)10	10	153347	153356	IRB
Epimedium mikinorii_GZLS						
1	p1	(A)11	11	259	269	LSC
2	p1	(A)11	11	4021	4031	LSC
3	p1	(A)10	10	4857	4866	LSC
4	p1	(T)10	10	5599	5608	LSC

5	p1	(T)11	11	6530	6540	LSC
6	p1	(T)10	10	7463	7472	LSC
7	p1	(A)10	10	8036	8045	LSC
8	p1	(A)11	11	8584	8594	LSC
9	p1	(T)12	12	8612	8623	LSC
10	p1	(A)12	12	8815	8826	LSC
11	p1	(A)10	10	13025	13034	LSC
12	p1	(A)10	10	13683	13692	LSC
13	p5	(AGATA)3	15	14779	14793	LSC
14	p1	(A)14	14	15068	15081	LSC
15	p1	(T)10	10	18856	18865	LSC
16	p1	(T)11	11	22995	23005	LSC
17	p1	(T)10	10	26644	26653	LSC
18	p1	(T)10	10	29556	29565	LSC
19	p1	(T)10	10	30257	30266	LSC
20	p1	(T)12	12	31684	31695	LSC
21	p1	(G)11	11	35482	35492	LSC
22	p1	(A)15	15	38499	38513	LSC
23	p1	(C)10	10	41471	41480	LSC
24	p1	(T)10	10	43104	43113	LSC
25	p3	(ATA)4	12	43327	43338	LSC
26	p1	(T)10	10	44031	44040	LSC
27	p1	(T)10	10	45525	45534	LSC
28	p1	(A)14	14	45593	45606	LSC
29	p1	(A)11	11	45852	45862	LSC
30	p1	(T)11	11	47607	47617	LSC
31	p2	(TA)6	12	49902	49913	LSC
32	p1	(T)10	10	50537	50546	LSC
33	p1	(T)12	12	54001	54012	LSC
34	p1	(T)10	10	54133	54142	LSC
35	p1	(A)12	12	57564	57575	LSC
36	p3	(GGA)4	12	60675	60686	LSC

37	p1	(A)19	19	61852	61870	LSC
38	p1	(T)11	11	63196	63206	LSC
39	p1	(A)11	11	65251	65261	LSC
40	p1	(T)12	12	65404	65415	LSC
41	p1	(T)12	12	65612	65623	LSC
42	p1	(A)10	10	66224	66233	LSC
43	p1	(A)10	10	66853	66862	LSC
44	p1	(T)15	15	66922	66936	LSC
45	p1	(A)11	11	68793	68803	LSC
46	p1	(T)16	16	68938	68953	LSC
47	p1	(T)10	10	71969	71978	LSC
48	p1	(A)13	13	72291	72303	LSC
49	p1	(A)13	13	72838	72850	LSC
50	p1	(T)14	14	73001	73014	LSC
51	p1	(T)11	11	73738	73748	LSC
52	p1	(T)11	11	73951	73961	LSC
53	p1	(T)11	11	77811	77821	LSC
54	p1	(A)11	11	78051	78061	LSC
55	p1	(T)12	12	78185	78196	LSC
56	p1	(T)10	10	82726	82735	LSC
57	p1	(T)14	14	83275	83288	LSC
58	p1	(A)13	13	84384	84396	LSC
59	p1	(T)12	12	85004	85015	LSC
60	p1	(T)10	10	85092	85101	LSC
61	p1	(T)17	17	85157	85173	LSC
62	p1	(T)11	11	85263	85273	LSC
63	p1	(T)10	10	85541	85550	LSC
64	p1	(A)10	10	92131	92140	IRA
65	p1	(A)13	13	112807	112819	IRA
66	p3	(AGG)4	12	114207	114218	IRA
67	p1	(T)14	14	116803	116816	SSC
68	p1	(A)10	10	116931	116940	SSC

69	p1	(T)11	11	117328	117338	SSC
70	p2	(AT)7	14	117548	117561	SSC
71	p1	(T)14	14	117671	117684	SSC
72	p1	(A)13	13	124509	124521	SSC
73	p1	(A)13	13	131048	131060	IRB
74	p3	(TCC)4	12	131224	131235	IRB
75	p1	(T)13	13	132624	132636	IRB
76	p1	(T)10	10	153303	153312	IRB
Epimedium pseudowushanense_GZNW						
1	p1	(A)11	11	259	269	LSC
2	p1	(A)11	11	4028	4038	LSC
3	p1	(A)11	11	4864	4874	LSC
4	p1	(T)10	10	5607	5616	LSC
5	p1	(T)11	11	6538	6548	LSC
6	p1	(T)10	10	7471	7480	LSC
7	p1	(A)10	10	8035	8044	LSC
8	p1	(A)12	12	8583	8594	LSC
9	p1	(T)11	11	8612	8622	LSC
10	p1	(A)13	13	8814	8826	LSC
11	p1	(A)10	10	13016	13025	LSC
12	p1	(A)10	10	13674	13683	LSC
13	p5	(AGATA)3	15	14770	14784	LSC
14	p1	(A)14	14	15059	15072	LSC
15	p1	(T)10	10	18847	18856	LSC
16	p1	(T)11	11	22986	22996	LSC
17	p1	(T)10	10	26635	26644	LSC
18	p1	(T)10	10	29572	29581	LSC
19	p1	(T)10	10	30273	30282	LSC
20	p1	(T)12	12	31700	31711	LSC
21	p1	(G)11	11	35498	35508	LSC
22	p1	(A)16	16	38512	38527	LSC
23	p1	(C)10	10	41485	41494	LSC

24	p1	(T)10	10	43129	43138	LSC
25	p3	(ATA)4	12	43352	43363	LSC
26	p1	(T)10	10	44056	44065	LSC
27	p1	(T)10	10	45550	45559	LSC
28	p1	(A)14	14	45618	45631	LSC
29	p1	(A)11	11	45877	45887	LSC
30	p1	(T)10	10	47632	47641	LSC
31	p2	(TA)6	12	49920	49931	LSC
32	p1	(T)10	10	50555	50564	LSC
33	p1	(T)13	13	54020	54032	LSC
34	p1	(T)10	10	54153	54162	LSC
35	p1	(A)13	13	57584	57596	LSC
36	p3	(GGA)4	12	60708	60719	LSC
37	p1	(A)18	18	61879	61896	LSC
38	p1	(T)12	12	63222	63233	LSC
39	p1	(A)10	10	65278	65287	LSC
40	p1	(T)11	11	65430	65440	LSC
41	p1	(T)12	12	65637	65648	LSC
42	p1	(A)10	10	66249	66258	LSC
43	p1	(A)10	10	66878	66887	LSC
44	p1	(T)15	15	66947	66961	LSC
45	p1	(A)12	12	68818	68829	LSC
46	p1	(T)16	16	68964	68979	LSC
47	p1	(T)11	11	71995	72005	LSC
48	p1	(A)13	13	72318	72330	LSC
49	p1	(A)13	13	72865	72877	LSC
50	p1	(T)13	13	73028	73040	LSC
51	p1	(T)10	10	73764	73773	LSC
52	p1	(T)11	11	73976	73986	LSC
53	p1	(T)11	11	77836	77846	LSC
54	p1	(A)10	10	78076	78085	LSC
55	p1	(T)11	11	78209	78219	LSC

56	p1	(T)10	10	82749	82758	LSC
57	p1	(T)13	13	83298	83310	LSC
58	p1	(A)13	13	84406	84418	LSC
59	p1	(T)12	12	85026	85037	LSC
60	p1	(T)12	12	85179	85190	LSC
61	p1	(T)11	11	85279	85289	LSC
62	p1	(T)10	10	85557	85566	LSC
63	p1	(A)10	10	92153	92162	IRA
64	p1	(A)12	12	112829	112840	IRA
65	p3	(AGG)4	12	114228	114239	IRA
66	p6	(CAACGA)3	18	114228	114245	IRA
67	p1	(T)14	14	116824	116837	SSC
68	p1	(A)10	10	116952	116961	SSC
69	p1	(T)11	11	117591	117601	SSC
70	p2	(AT)7	14	117811	117824	SSC
71	p1	(T)15	15	117934	117948	SSC
72	p1	(A)14	14	124773	124786	SSC
73	p1	(T)11	11	127557	127567	SSC
74	p1	(A)13	13	131315	131327	IRB
75	p3	(TCC)4	12	131491	131502	IRB
76	p1	(T)12	12	132891	132902	IRB
77	p1	(T)10	10	153569	153578	IRB
Epimedium wushanense_HBXS-2						
1	p1	(A)11	11	248	258	LSC
2	p1	(A)10	10	4010	4019	LSC
3	p1	(A)11	11	4845	4855	LSC
4	p1	(T)10	10	5588	5597	LSC
5	p1	(T)12	12	6521	6532	LSC
6	p1	(T)10	10	7455	7464	LSC
7	p1	(A)10	10	8019	8028	LSC
8	p1	(A)16	16	8562	8577	LSC
9	p1	(T)10	10	8591	8600	LSC

10	p1	(A)11	11	8792	8802	LSC
11	p1	(A)10	10	12987	12996	LSC
12	p1	(A)11	11	13652	13662	LSC
13	p1	(T)10	10	18815	18824	LSC
14	p1	(T)13	13	22954	22966	LSC
15	p1	(T)10	10	26605	26614	LSC
16	p2	(AT)6	12	27389	27400	LSC
17	p1	(A)10	10	28171	28180	LSC
18	p1	(T)10	10	29531	29540	LSC
19	p1	(T)10	10	30232	30241	LSC
20	p1	(T)12	12	31658	31669	LSC
21	p1	(G)11	11	35471	35481	LSC
22	p1	(A)15	15	38500	38514	LSC
23	p1	(C)10	10	41472	41481	LSC
24	p1	(T)10	10	43116	43125	LSC
25	p1	(A)10	10	43720	43729	LSC
26	p1	(T)10	10	44050	44059	LSC
27	p1	(T)10	10	44524	44533	LSC
28	p1	(T)10	10	45545	45554	LSC
29	p1	(A)12	12	45613	45624	LSC
30	p1	(A)11	11	45870	45880	LSC
31	p1	(T)10	10	47624	47633	LSC
32	p1	(T)10	10	48335	48344	LSC
33	p2	(TA)6	12	49914	49925	LSC
34	p1	(T)13	13	50556	50568	LSC
35	p1	(T)10	10	54158	54167	LSC
36	p1	(A)12	12	57589	57600	LSC
37	p3	(GGA)4	12	60713	60724	LSC
38	p1	(A)12	12	61918	61929	LSC
39	p1	(A)11	11	62203	62213	LSC
40	p1	(T)10	10	63257	63266	LSC
41	p1	(A)11	11	65311	65321	LSC

42	p1	(T)10	10	65464	65473	LSC
43	p1	(T)10	10	65670	65679	LSC
44	p1	(A)10	10	66280	66289	LSC
45	p1	(A)11	11	66909	66919	LSC
46	p1	(T)13	13	66980	66992	LSC
47	p1	(A)10	10	68287	68296	LSC
48	p1	(A)11	11	68858	68868	LSC
49	p1	(T)14	14	68997	69010	LSC
50	p1	(T)11	11	72022	72032	LSC
51	p1	(A)11	11	72344	72354	LSC
52	p1	(A)14	14	72887	72900	LSC
53	p1	(T)14	14	73051	73064	LSC
54	p1	(T)10	10	73782	73791	LSC
55	p1	(T)11	11	73994	74004	LSC
56	p1	(T)10	10	77854	77863	LSC
57	p1	(A)10	10	78099	78108	LSC
58	p1	(T)10	10	78232	78241	LSC
59	p1	(A)11	11	82809	82819	LSC
60	p1	(T)14	14	83320	83333	LSC
61	p1	(A)13	13	84430	84442	LSC
62	p1	(T)11	11	85064	85074	LSC
63	p1	(T)16	16	85216	85231	LSC
64	p1	(T)10	10	85320	85329	LSC
65	p1	(T)10	10	85597	85606	LSC
66	p1	(A)10	10	92187	92196	IRA
67	p1	(A)13	13	112863	112875	IRA
68	p3	(AGG)4	12	114263	114274	IRA
69	p1	(T)14	14	116861	116874	SSC
70	p1	(A)10	10	116925	116934	SSC
71	p1	(A)10	10	116990	116999	SSC
72	p1	(T)11	11	117630	117640	SSC
73	p2	(AT)8	16	117850	117865	SSC

74	p1	(T)16	16	117975	117990	SSC
75	p1	(A)13	13	124822	124834	SSC
76	p1	(A)16	16	131361	131376	IRB
77	p3	(TCC)4	12	131537	131548	IRB
78	p1	(T)13	13	132937	132949	IRB
79	p1	(T)10	10	153616	153625	IRB
Epimedium ilicifolium_SXBX						
1	p1	(A)12	12	4017	4028	LSC
2	p1	(A)12	12	4854	4865	LSC
3	p1	(T)10	10	5598	5607	LSC
4	p1	(T)10	10	6529	6538	LSC
5	p1	(T)10	10	7461	7470	LSC
6	p1	(A)11	11	8025	8035	LSC
7	p1	(A)10	10	8574	8583	LSC
8	p1	(T)11	11	8595	8605	LSC
9	p1	(A)12	12	8797	8808	LSC
10	p1	(A)11	11	13002	13012	LSC
11	p1	(A)10	10	13661	13670	LSC
12	p1	(A)14	14	15041	15054	LSC
13	p1	(T)10	10	18829	18838	LSC
14	p1	(T)12	12	22968	22979	LSC
15	p1	(T)10	10	26618	26627	LSC
16	p1	(A)11	11	28176	28186	LSC
17	p1	(T)10	10	29531	29540	LSC
18	p1	(T)11	11	30247	30257	LSC
19	p1	(T)12	12	31674	31685	LSC
20	p1	(G)11	11	35473	35483	LSC
21	p1	(A)11	11	38505	38515	LSC
22	p1	(C)10	10	41473	41482	LSC
23	p1	(T)10	10	43117	43126	LSC
24	p3	(ATA)4	12	43340	43351	LSC
25	p1	(A)13	13	43717	43729	LSC

26	p1	(T)12	12	44050	44061	LSC
27	p1	(T)10	10	44526	44535	LSC
28	p1	(T)11	11	45547	45557	LSC
29	p1	(A)13	13	45616	45628	LSC
30	p1	(A)11	11	45874	45884	LSC
31	p1	(T)10	10	47629	47638	LSC
32	p2	(TA)6	12	49909	49920	LSC
33	p1	(T)11	11	50551	50561	LSC
34	p4	(TAAA)4	16	52707	52722	LSC
35	p1	(T)11	11	54006	54016	LSC
36	p1	(T)10	10	54137	54146	LSC
37	p1	(A)13	13	57568	57580	LSC
38	p5	(ATTAT)3	15	59556	59570	LSC
39	p3	(GGA)4	12	60698	60709	LSC
40	p1	(A)14	14	61900	61913	LSC
41	p3	(AAG)4	12	62175	62186	LSC
42	p1	(T)11	11	63239	63249	LSC
43	p1	(T)10	10	65650	65659	LSC
44	p1	(A)10	10	66260	66269	LSC
45	p1	(A)12	12	66889	66900	LSC
46	p1	(T)13	13	66961	66973	LSC
47	p1	(A)13	13	68261	68273	LSC
48	p1	(A)11	11	68829	68839	LSC
49	p1	(T)14	14	68974	68987	LSC
50	p1	(T)11	11	72004	72014	LSC
51	p1	(A)13	13	72289	72301	LSC
52	p1	(A)13	13	72836	72848	LSC
53	p1	(T)14	14	72999	73012	LSC
54	p1	(T)11	11	73730	73740	LSC
55	p1	(T)11	11	73943	73953	LSC
56	p1	(T)10	10	77803	77812	LSC
57	p1	(A)10	10	78042	78051	LSC

58	p1	(T)12	12	78175	78186	LSC
59	p1	(T)10	10	82715	82724	LSC
60	p1	(T)15	15	83264	83278	LSC
61	p1	(A)14	14	84375	84388	LSC
62	p1	(T)12	12	84709	84720	LSC
63	p1	(T)10	10	84959	84968	LSC
64	p1	(T)10	10	85236	85245	LSC
65	p1	(A)10	10	91832	91841	IRA
66	p1	(A)13	13	112519	112531	IRA
67	p3	(AGG)4	12	113919	113930	IRA
68	p1	(A)11	11	116624	116634	SSC
69	p1	(T)11	11	117255	117265	SSC
70	p2	(AT)6	12	117475	117486	SSC
71	p1	(T)14	14	117596	117609	SSC
72	p1	(A)13	13	124420	124432	SSC
73	p1	(A)13	13	130965	130977	IRB
74	p3	(TCC)4	12	131141	131152	IRB
75	p1	(T)13	13	132541	132553	IRB
76	p1	(T)10	10	153231	153240	IRB

Table S4. Repeated sequences identified in the cp genomes of *E. wushanense* and its closely related species.

Genomes /SSR No.	Repeat Type	Length (bp)	Position A	Locus	Region	Position B	Locus	Regio n
*Epimedium_wushanense*_HBXS								
1	P	131	5830	*trnK-UUU-rps16*	LSC	103594	*trnV-GAC-rrn16*	IRA
2	F	131	5830	*trnK-UUU-rps16*	LSC	141995	*rrn16*	IRB
3	F	107	4635	*trnK-UUU*	LSC	109596	*rrn23*	IRA
4	P	107	4635	*trnK-UUU*	LSC	136017	*rrn23*	IRB
5	P	71	62228	*accD-psaI*	LSC	62228	*accD-psaI*	LSC
6	F	77	4745	*trnK-UUU*	LSC	109708	*rrn23*	IRA
7	P	77	4745	*trnK-UUU*	LSC	135935	*rrn23*	IRB

8	F	77	114004	*ycf1*	IRA	114118	*ycf1*	IRA
9	P	77	114004	*ycf1*	IRA	131525	*ycf1*	IRB
10	P	77	114118	*ycf1*	IRA	131639	*ycf1*	IRB
11	F	77	131525	*ycf1*	IRB	131639	*ycf1*	IRB
12	F	62	131545	*ycf1*	IRB	131659	*ycf1*	IRB
13	F	53	4689	*trnK-UUU*	LSC	109650	*rrn23*	IRA
14	P	53	4689	*trnK-UUU*	LSC	136017	*rrn23*	IRB
15	P	48	54750	*trnV-UAC-trnM-CAU*	LSC	54750	*trnV-UAC-trnM-CAU*	LSC
16	F	53	113997	*ycf1*	IRA	114063	*ycf1*	IRA
17	P	53	113997	*ycf1*	IRA	131604	*ycf1*	IRB
18	P	53	114063	*ycf1*	IRA	131670	*ycf1*	IRB
19	F	53	131604	*ycf1*	IRB	131670	*ycf1*	IRB
20	F	49	4773	*trnK-UUU*	LSC	109736	*rrn23*	IRA
21	P	49	4773	*trnK-UUU*	LSC	135935	*rrn23*	IRB
22	P	49	98018	*trnL-CAA-ndhB*	IRA	98018	*trnL-CAA-ndhB*	IRA
23	F	49	98018	*trnL-CAA-ndhB*	IRA	147653	*ndhB-trnL-CAA*	IRB
24	P	49	147653	*ndhB-trnL-CAA*	IRB	147653	*ndhB-trnL-CAA*	IRB
25	P	44	29974	*petN-psbM*	LSC	29974	*petN-psbM*	LSC
26	F	50	74370	*clpP*	LSC	74418	*clpP*	LSC
27	F	46	114070	*ycf1*	IRA	114118	*ycf1*	IRA
28	P	46	114070	*ycf1*	IRA	131556	*ycf1*	IRB
29	P	46	114118	*ycf1*	IRA	131604	*ycf1*	IRB
30	F	46	131556	*ycf1*	IRB	131604	*ycf1*	IRB
31	F	37	92083	*ycf2*	IRA	92140	*ycf2*	IRA
32	P	37	92083	*ycf2*	IRA	153543	*ycf2*	IRB
33	P	37	92140	*ycf2*	IRA	153600	*ycf2*	IRB
34	F	37	153543	*ycf2*	IRB	153600	*ycf2*	IRB
35	F	40	131617	*ycf1*	IRB	131683	*ycf1*	IRB
36	P	35	5926	*trnK-UUU-rps16*	LSC	103594	*trnV-GAC-rrn16*	IRA
37	F	35	5926	*trnK-UUU-rps16*	LSC	142091	*rrn16-trnV-GAC*	IRB
38	P	35	65749	*petA-psbJ*	LSC	65791	*petA-psbJ*	LSC
39	F	44	94749	*ycf2*	IRA	94758	*ycf2*	IRA

40	P	44	94749	*ycf2*	IRA	150918	*ycf2*	IRB
41	P	44	94758	*ycf2*	IRA	150927	*ycf2*	IRB
42	F	44	150918	*ycf2*	IRB	150927	*ycf2*	IRB
43	P	43	49687	*trnL-UAA-trnF-GAA*	LSC	49687	*trnL-UAA-trnF-GAA*	LSC
44	F	43	113915	*ycf1*	IRA	113927	*ycf1*	IRA
45	P	43	113915	*ycf1*	IRA	131750	*ycf1*	IRB
46	P	43	113927	*ycf1*	IRA	131762	*ycf1*	IRB
47	F	43	131750	*ycf1*	IRB	131762	*ycf1*	IRB
48	P	33	36465	*psbC-trnS-UGA*	LSC	36510	*psbC-trnS-UGA*	LSC
49	F	33	131569	*ycf1*	IRB	131617	*ycf1*	IRB
*Epimedium_borealiguizhouense*_GZQB								
1	F	189	4627	*trnK-UUU-rps16*	LSC	109682	*rrn23*	IRA
2	P	189	4627	*trnK-UUU-rps16*	LSC	136012	*rrn23*	IRB
3	F	135	4681	*trnK-UUU-rps16*	LSC	109736	*rrn23*	IRA
4	P	135	4681	*trnK-UUU-rps16*	LSC	136012	*rrn23*	IRB
5	P	131	5821	*trnK-UUU-rps16*	LSC	103680	*trnV-GAC-rrn16*	IRA
6	F	131	5821	*trnK-UUU-rps16*	LSC	142072	*rrn16*	IRB
7	F	82	114085	*ycf1*	IRA	114199	*ycf1*	IRA
8	P	82	114085	*ycf1*	IRA	131602	*ycf1*	IRB
9	P	82	114199	*ycf1*	IRA	131716	*ycf1*	IRB
10	F	82	131602	*ycf1*	IRB	131716	*ycf1*	IRB
11	P	71	62230	*accD-psaI*	LSC	62230	*accD-psaI*	LSC
12	F	62	131622	*ycf1*	IRB	131736	*ycf1*	IRB
13	P	48	54757	*trnV-UAC-trnM-CAU*	LSC	54757	*trnV-UAC-trnM-CAU*	LSC
14	F	53	114083	*ycf1*	IRA	114149	*ycf1*	IRA
15	P	53	114083	*ycf1*	IRA	131681	*ycf1*	IRB
16	P	53	114149	*ycf1*	IRA	131747	*ycf1*	IRB
17	F	53	131681	*ycf1*	IRB	131747	*ycf1*	IRB
18	P	49	98095	*trnL-CAA-ndhB*	IRA	98095	*trnL-CAA-ndhB*	IRA
19	F	49	98095	*trnL-CAA-ndhB*	IRA	147739	*ndhB-trnL-CAA*	IRB
20	P	49	147739	*ndhB-trnL-CAA*	IRB	147739	*ndhB-trnL-CAA*	IRB
21	P	44	29955	*petN-psbM*	LSC	29955	*petN-psbM*	LSC

22	F	47	72262	*rps12-clpP*	LSC	72307	*rps12-clpP*	LSC
23	F	50	74449	*clpP_extron_2-clpP_extron_1*	*clpP_extron_2-clpP_extron_1*	74497	*clpP_extron_2-clpP_extron_1*	LSC
24	F	46	114156	*ycf1*	IRA	114204	*ycf1*	IRA
25	P	46	114156	*ycf1*	IRA	131633	*ycf1*	IRB
26	P	46	114204	*ycf1*	IRA	131681	*ycf1*	IRB
27	F	46	131633	*ycf1*	IRB	131681	*ycf1*	IRB
28	F	37	92163	*ycf2*	IRA	92220	*ycf2*	IRA
29	P	37	92163	*ycf2*	IRA	153626	*ycf2*	IRB
30	P	37	92220	*ycf2*	IRA	153683	*ycf2*	IRB
31	F	37	153626	*ycf2*	IRB	153683	*ycf2*	IRB
32	F	40	131694	*ycf1*	IRB	131760	*ycf1*	IRB
33	P	36	36446	*psbC-trnS-UGA*	LSC	36494	*psbC-trnS-UGA*	LSC
34	P	35	5917	*trnK-UUU-rps16*	LSC	103680	*trnV-GAC-rrn16*	IRA
35	F	35	5917	*trnK-UUU-rps16*	LSC	142168	*rrn16-trnV-GAC*	IRB
36	P	35	65754	*petA-psbJ*	LSC	65796	*petA-psbJ*	LSC
37	P	43	49674	*trnL-UAA-trnF-GAA*	LSC	49674	*trnL-UAA-trnF-GAA*	LSC
38	F	43	114001	*ycf1*	IRA	114013	*ycf1*	IRA
39	P	43	114001	*ycf1*	IRA	131827	*ycf1*	IRB
40	P	43	114013	*ycf1*	IRA	131839	*ycf1*	IRB
41	F	43	131827	*ycf1*	IRB	131839	*ycf1*	IRB
42	F	33	131646	*ycf1*	IRB	131694	*ycf1*	IRB
43	F	39	44938	*ycf3_extron_2-ycf3_extron_1*	LSC	102112	*rps12-trnV-GAC*	IRA
44	P	39	44938	*ycf3_extron_2-ycf3_extron_1*	LSC	143732	*trnV-GAC-rps12*	IRB
45	P	36	8626	*psbI-trnS-GCU*	LSC	46676	*trnS-GGA*	LSC
46	F	32	5497	*trnK-UUU-rps16*	LSC	5647	*trnK-UUU-rps16*	LSC
47	P	32	62141	*accD-psaI*	LSC	62180	*accD-psaI*	LSC
48	F	40	5534	*trnK-UUU-rps16*	LSC	5679	*trnK-UUU-rps16*	LSC
49	F	40	60681	*accD*	LSC	60756	*accD*	LSC

*Epimedium_chlorandrum*_SCMP

1	P	131	5832	*trnK-UUU-rps16*	LSC	103638	*trnV-GAC-rrn16*	IRA
2	F	131	5832	*trnK-UUU-rps16*	LSC	141108	*rrn16*	IRB
3	F	107	4635	*trnK-UUU-rps16*	LSC	109640	*rrn23*	IRA
4	P	107	4635	*trnK-UUU-rps16*	LSC	135130	*rrn23*	IRB
5	F	82	114004	*ycf1*	IRA	114118	*ycf1*	IRA
6	P	82	114004	*ycf1*	IRA	130677	*ycf1*	IRB
7	F	40	130769	*ycf1*	IRB	130835	*ycf1*	IRB
8	P	35	5928	*trnK-UUU-rps16*	LSC	103638	*trnV-GAC-rrn16*	IRA
9	F	43	113920	*ycf1*	IRA	113932	*ycf1*	IRA
10	P	43	113932	*ycf1*	IRA	130914	*ycf1*	IRB
11	P	53	114068	*ycf1*	IRA	130822	*ycf1*	IRB
12	F	43	130902	*ycf1*	IRB	130914	*ycf1*	IRB
13	P	48	54771	*trnV-UAC-trnM-CAU*	LSC	54771	*trnV-UAC-trnM-CAU*	LSC
14	P	46	114123	*ycf1*	IRA	130756	*ycf1*	IRB
15	F	46	130708	*ycf1*	IRB	130756	*ycf1*	IRB
16	F	35	5928	*trnK-UUU-rps16*	LSC	141204	*rrn16-trnV-GAC*	IRB
17	P	39	44954	*ycf3_extron_2-ycf3_extron_1*	LSC	142759	*trnV-GAC-rps12*	IRB
18	P	44	29969	*petN-psbM*	LSC	29969	*petN-psbM*	LSC
19	F	47	72295	*rps12-clpP*	LSC	72340	*rps12-clpP*	LSC
20	F	40	60715	*accD*	LSC	60790	*accD*	LSC
21	F	77	4745	*trnK-UUU-rps16*	LSC	109752	*rrn23*	IRA
22	F	49	4773	*trnK-UUU-rps16*	LSC	109780	*rrn23*	IRA
23	P	49	4773	*trnK-UUU-rps16*	LSC	135048	*rrn23*	IRB
24	P	35	65787	*petA-psbJ*	LSC	65829	*petA-psbJ*	LSC
25	P	43	49689	*trnL-UAA-trnF-GAA*	LSC	49689	*trnL-UAA-trnF-GAA*	LSC
26	P	43	113920	*ycf1*	IRA	130902	*ycf1*	IRB
27	F	33	130721	*ycf1*	IRB	130769	*ycf1*	IRB
28	P	49	98053	*trnL-CAA-ndhB*	IRA	98053	*trnL-CAA-ndhB*	IRA
29	F	49	98053	*trnL-CAA-ndhB*	IRA	146775	*ndhB-trnL-CAA*	IRB

30	P	49	146775	*ndhB-trnL-CAA*	IRB	146775	*ndhB-trnL-CAA*	IRB
31	P	34	27922	*rpoB-trnC-GCA*	LSC	28634	*trnC-GCA-petN*	LSC
32	P	36	8645	*psbI-trnS-GCU*	LSC	46691	*trnS-GGA*	LSC
33	P	77	4745	*trnK-UUU-rps16*	LSC	135048	*rrn23*	IRB
34	P	82	114118	*ycf1*	IRA	130791	*ycf1*	IRB
35	F	82	130677	*ycf1*	IRB	130791	*ycf1*	IRB
36	F	53	130756	*ycf1*	IRB	130822	*ycf1*	IRB
37	P	53	4689	*trnK-UUU-rps16*	LSC	135130	*rrn23*	IRB
38	F	50	74478	*clpP_extron_2-clpP_extron_1*	LSC	74526	*clpP_extron_2-clpP_extron_1*	LSC
39	F	46	114075	*ycf1*	IRA	114123	*ycf1*	IRA
40	P	46	114075	*ycf1*	IRA	130708	*ycf1*	IRB
41	F	32	5508	*trnK-UUU-rps16*	LSC	5658	*trnK-UUU-rps16*	LSC
42	F	39	44954	*ycf3_extron_2-ycf3_extron_1*	LSC	102079	*rps12-trnV-GAC*	IRA
43	F	62	130697	*ycf1*	IRB	130811	*ycf1*	IRB
44	F	53	4689	*trnK-UUU-rps16*	LSC	109694	*rrn23*	IRA
45	P	71	62265	*accD-psaI*	LSC	62265	*accD-psaI*	LSC
46	P	78	5885	*trnK-UUU-rps16*	LSC	103638	*trnV-GAC-rrn16*	IRA
47	P	53	114002	*ycf1*	IRA	130756	*ycf1*	IRB
48	F	78	5885	*trnK-UUU-rps16*	LSC	141161	*rrn16*	IRB
49	F	53	114002	*ycf1*	IRA	114068	*ycf1*	IRA
*Epimedium_pseudowushanense*_GZJH								
1	P	131	5839	*trnK-UUU-rps16*	LSC	103507	*trnV-GAC-rrn16*	IRA
2	F	131	5839	*trnK-UUU-rps16*	LSC	141887	*rrn16*	IRB
3	F	107	4641	*trnK-UUU-rps16*	LSC	109509	*rrn23*	IRA
4	P	107	4641	*trnK-UUU-rps16*	LSC	135909	*rrn23*	IRB
5	F	82	113912	*ycf1*	IRA	114026	*ycf1*	IRA
6	P	82	113912	*ycf1*	IRA	131417	*ycf1*	IRB
7	P	82	114026	*ycf1*	IRA	131531	*ycf1*	IRB
8	F	82	131417	*ycf1*	IRB	131531	*ycf1*	IRB
9	P	71	62088	*accD-psaI*	LSC	62088	*accD-psaI*	LSC

10	F	77	4751	*trnK-UUU-rps16*	LSC	109621	*rrn23*	IRA
11	P	77	4751	*trnK-UUU-rps16*	LSC	135827	*rrn23*	IRB
12	F	69	5509	*trnK-UUU-rps16*	LSC	5660	*trnK-UUU-rps16*	LSC
13	F	62	131437	*ycf1*	IRB	131551	*ycf1*	IRB
14	F	53	4695	*trnK-UUU-rps16*	LSC	109563	*rrn23*	IRA
15	P	53	4695	*trnK-UUU-rps16*	LSC	135909	*rrn23*	IRB
16	P	48	54645	*trnV-UAC-trnM-CAU*	LSC	54645	*trnV-UAC-trnM-CAU*	LSC
17	F	53	113910	*ycf1*	IRA	113976	*ycf1*	IRA
18	P	53	113910	*ycf1*	IRA	131496	*ycf1*	IRB
19	P	53	113976	*ycf1*	IRA	131562	*ycf1*	IRB
20	F	53	131496	*ycf1*	IRB	131562	*ycf1*	IRB
21	F	49	4779	*trnK-UUU-rps16*	LSC	109649	*rrn23*	IRA
22	P	49	4779	*trnK-UUU-rps16*	LSC	135827	*rrn23*	IRB
23	P	49	97931	*trnL-CAA-ndhB*	IRA	97931	*trnL-CAA-ndhB*	IRA
24	F	49	97931	*trnL-CAA-ndhB*	IRA	147545	*ndhB-trnL-CAA*	IRB
25	P	49	147545	*ndhB-trnL-CAA*	IRB	147545	*ndhB-trnL-CAA*	IRB
26	P	44	29873	*petN-psbM*	LSC	29873	*petN-psbM*	LSC
27	F	47	72099	*rps12-clpP*	LSC	72144	*rps12-clpP*	LSC
28	F	50	74291	*clpP_extron_2-clpP_extron_1*	LSC	74339	*clpP_extron_2-clpP_extron_1*	LSC
29	F	46	113983	*ycf1*	IRA	114031	*ycf1*	IRA
30	P	46	113983	*ycf1*	IRA	131448	*ycf1*	IRB
31	P	46	114031	*ycf1*	IRA	131496	*ycf1*	IRB
32	F	46	131448	*ycf1*	IRB	131496	*ycf1*	IRB
33	F	37	92009	*ycf2*	IRA	92066	*ycf2*	IRA
34	P	37	92009	*ycf2*	IRA	153422	*ycf2*	IRB
35	P	37	92066	*ycf2*	IRA	153479	*ycf2*	IRB
36	F	37	153422	*ycf2*	IRB	153479	*ycf2*	IRB
37	F	40	131509	*ycf1*	IRB	131575	*ycf1*	IRB
38	F	43	5568	*trnK-UUU-rps16*	LSC	5729	*trnK-UUU-rps16*	LSC
39	P	35	5935	*trnK-UUU-rps16*	LSC	103507	*trnV-GAC-rrn16*	IRA
40	F	35	5935	*trnK-UUU-rps16*	LSC	141983	*rrn16-trnV-GAC*	IRB

41	P	35	65616	*petA-psbJ*	LSC	65658	*petA-psbJ*	LSC
42	P	43	49580	*trnL-UAA-trnF-GAA*	LSC	49580	*trnL-UAA-trnF-GAA*	LSC
43	F	43	113828	*ycf1*	IRA	113840	*ycf1*	IRA
44	P	43	113828	*ycf1*	IRA	131642	*ycf1*	IRB
45	P	43	113840	*ycf1*	IRA	131654	*ycf1*	IRB
46	F	43	131642	*ycf1*	IRB	131654	*ycf1*	IRB
47	F	33	131461	*ycf1*	IRB	131509	*ycf1*	IRB
48	F	39	44839	*ycf3_extron_2-ycf3_extron_1*	LSC	101948	*rps12-trnV-GAC*	IRA
49	P	39	44839	*ycf3_extron_2-ycf3_extron_1*	LSC	143538	*trnV-GAC-rps12*	IRB
*Epimedium_mikinorii*_HBES								
1	P	131	5837	*trnK-UUU-rps16*	LSC	103414	*trnV-GAC-rrn16*	IRA
2	F	131	5837	*trnK-UUU-rps16*	LSC	141798	*rrn16*	IRB
3	F	107	4626	*trnK-UUU-rps16*	LSC	109416	*rrn23*	IRA
4	P	107	4626	*trnK-UUU-rps16*	LSC	135820	*rrn23*	IRB
5	F	82	113819	*ycf1*	IRA	113933	*ycf1*	IRA
6	P	82	113819	*ycf1*	IRA	131328	*ycf1*	IRB
7	P	82	113933	*ycf1*	IRA	131442	*ycf1*	IRB
8	F	82	131328	*ycf1*	IRB	131442	*ycf1*	IRB
9	P	71	62153	*accD-psaI*	LSC	62153	*accD-psaI*	LSC
10	F	72	4761	*trnK-UUU-rps16*	LSC	109533	*rrn23*	IRA
11	P	72	4761	*trnK-UUU-rps16*	LSC	135738	*rrn23*	IRB
12	F	62	131348	*ycf1*	IRB	131462	*ycf1*	IRB
13	F	53	4680	*trnK-UUU-rps16*	LSC	109470	*rrn23*	IRA
14	P	53	4680	*trnK-UUU-rps16*	LSC	135820	*rrn23*	IRB
15	P	48	54684	*trnV-UAC-trnM-CAU*	LSC	54684	*trnV-UAC-trnM-CAU*	LSC
16	F	53	113817	*ycf1*	IRA	113883	*ycf1*	IRA
17	P	53	113817	*ycf1*	IRA	131407	*ycf1*	IRB
18	P	53	113883	*ycf1*	IRA	131473	*ycf1*	IRB
19	F	53	131407	*ycf1*	IRB	131473	*ycf1*	IRB
20	F	49	4784	*trnK-UUU-rps16*	LSC	109556	*rrn23*	IRA

21	P	49	4784	*trnK-UUU-rps16*	LSC	135738	*rrn23*	IRB
22	P	49	97838	*trnL-CAA-ndhB*	IRA	97838	*trnL-CAA-ndhB*	IRA
23	F	49	97838	*trnL-CAA-ndhB*	IRA	147456	*ndhB-trnL-CAA*	IRB
24	P	49	147456	*ndhB-trnL-CAA*	IRB	147456	*ndhB-trnL-CAA*	IRB
25	P	44	29980	*petN-psbM*	LSC	29980	*petN-psbM*	LSC
26	F	47	72109	*rps12-clpP*	LSC	72154	*rps12-clpP*	LSC
27	F	50	74292	*clpP_extron_2-clpP_extron_1*	LSC	74340	*clpP_extron_2-clpP_extron_1*	LSC
28	F	46	113890	*ycf1*	IRA	113938	*ycf1*	IRA
29	P	46	113890	*ycf1*	IRA	131359	*ycf1*	IRB
30	P	46	113938	*ycf1*	IRA	131407	*ycf1*	IRB
31	F	46	131359	*ycf1*	IRB	131407	*ycf1*	IRB
32	F	40	131420	*ycf1*	IRB	131486	*ycf1*	IRB
33	P	36	36454	*psbC-trnS-UGA*	LSC	36502	*psbC-trnS-UGA*	LSC
34	P	35	5933	*trnK-UUU-rps16*	LSC	103414	*trnV-GAC-rrn16*	IRA
35	F	35	5933	*trnK-UUU-rps16*	LSC	141894	*rrn16-trnV-GAC*	IRB
36	P	35	65674	*petA-psbJ*	LSC	65716	*petA-psbJ*	LSC
37	P	43	49632	*trnL-UAA-trnF-GAA*	LSC	49632	*trnL-UAA-trnF-GAA*	LSC
38	F	43	113735	*ycf1*	IRA	113747	*ycf1*	IRA
39	P	43	113735	*ycf1*	IRA	131553	*ycf1*	IRB
40	P	43	113747	*ycf1*	IRA	131565	*ycf1*	IRB
41	F	43	131553	*ycf1*	IRB	131565	*ycf1*	IRB
42	F	33	131372	*ycf1*	IRB	131420	*ycf1*	IRB
43	F	39	44898	*ycf3_extron_2-ycf3_extron_1*	LSC	101855	*rps12-trnV-GAC*	IRA
44	P	39	44898	*ycf3_extron_2-ycf3_extron_1*	LSC	143449	*trnV-GAC-rps12*	IRB
45	P	36	8656	*psbI-trnS-GCU*	LSC	46634	*trnS-GGA*	LSC
46	F	32	5513	*trnK-UUU-rps16*	LSC	5663	*trnK-UUU-rps16*	LSC
47	F	40	60613	*accD*	LSC	60688	*accD*	LSC
48	P	34	27932	*rpoB-trnC-GCA*	LSC	28645	*trnC-GCA-petN*	LSC

49	F	33	74309	*clpP_extron_2-clpP_extron_1*	LSC	74357	*clpP_extron_2-clpP_extron_1*	LSC
*Epimedium_wushanense*_HBXS_2								
1	P	131	5819	*trnK-UUU-rps16*	LSC	103640	*trnV-GAC-rrn16*	IRA
2	F	131	5819	*trnK-UUU-rps16*	LSC	142040	*rrn16*	IRB
3	F	107	4625	*trnK-UUU-rps16*	LSC	109642	*rrn23*	IRA
4	P	107	4625	*trnK-UUU-rps16*	LSC	136062	*rrn23*	IRB
5	P	71	62219	*accD-psaI*	LSC	62219	*accD-psaI*	LSC
6	F	77	4735	*trnK-UUU-rps16*	LSC	109754	*rrn23*	IRA
7	P	77	4735	*trnK-UUU-rps16*	LSC	135980	*rrn23*	IRB
8	F	77	114050	*ycf1*	IRA	114164	*ycf1*	IRA
9	P	77	114050	*ycf1*	IRA	131570	*ycf1*	IRB
10	P	77	114164	*ycf1*	IRA	131684	*ycf1*	IRB
11	F	77	131570	*ycf1*	IRB	131684	*ycf1*	IRB
12	F	62	131590	*ycf1*	IRB	131704	*ycf1*	IRB
13	F	53	4679	*trnK-UUU-rps16*	LSC	109696	*rrn23*	IRA
14	P	53	4679	*trnK-UUU-rps16*	LSC	136062	*rrn23*	IRB
15	P	48	54746	*trnV-UAC-trnM-CAU*	LSC	54746	*trnV-UAC-trnM-CAU*	LSC
16	F	53	114043	*ycf1*	IRA	114109	*ycf1*	IRA
17	P	53	114043	*ycf1*	IRA	131649	*ycf1*	IRB
18	P	53	114109	*ycf1*	IRA	131715	*ycf1*	IRB
19	F	53	131649	*ycf1*	IRB	131715	*ycf1*	IRB
20	F	49	4763	*trnK-UUU-rps16*	LSC	109782	*rrn23*	IRA
21	P	49	4763	*trnK-UUU-rps16*	LSC	135980	*rrn23*	IRB
22	P	49	98064	*trnL-CAA-ndhB*	IRA	98064	*trnL-CAA-ndhB*	IRA
23	F	49	98064	*trnL-CAA-ndhB*	IRA	147698	*ndhB-trnL-CAA*	IRB
24	P	49	147698	*ndhB-trnL-CAA*	IRB	147698	*ndhB-trnL-CAA*	IRB
25	P	44	29945	*petN-psbM*	LSC	29945	*petN-psbM*	LSC
26	F	47	72231	*rps12-clpP*	LSC	72276	*rps12-clpP*	LSC
27	F	50	74413	*clpP_extron_2-clpP_extron_1*	LSC	74461	*clpP_extron_2-clpP_extron_1*	LSC

28	F	46	114116	*ycf1*	IRA	114164	*ycf1*	IRA
29	P	46	114116	*ycf1*	IRA	131601	*ycf1*	IRB
30	P	46	114164	*ycf1*	IRA	131649	*ycf1*	IRB
31	F	46	131601	*ycf1*	IRB	131649	*ycf1*	IRB
32	F	37	92138	*ycf2*	IRA	92195	*ycf2*	IRA
33	P	37	92138	*ycf2*	IRA	153579	*ycf2*	IRB
34	P	37	92195	*ycf2*	IRA	153636	*ycf2*	IRB
35	F	37	153579	*ycf2*	IRB	153636	*ycf2*	IRB
36	F	40	131662	*ycf1*	IRB	131728	*ycf1*	IRB
37	P	35	5915	*trnK-UUU-rps16*	LSC	103640	*trnV-GAC-rrn16*	IRA
38	F	35	5915	*trnK-UUU-rps16*	LSC	142136	*rrn16-trnV-GAC*	IRB
39	P	35	65742	*petA-psbJ*	LSC	65784	*petA-psbJ*	LSC
40	P	43	49681	*trnL-UAA-trnF-GAA*	LSC	49681	*trnL-UAA-trnF-GAA*	LSC
41	F	43	113961	*ycf1*	IRA	113973	*ycf1*	IRA
42	P	43	113961	*ycf1*	IRA	131795	*ycf1*	IRB
43	P	43	113973	*ycf1*	IRA	131807	*ycf1*	IRB
44	F	43	131795	*ycf1*	IRB	131807	*ycf1*	IRB
45	P	33	36450	*psbC-trnS-UGA*	LSC	36495	*psbC-trnS-UGA*	LSC
46	F	33	131614	*ycf1*	IRB	131662	*ycf1*	IRB
47	F	39	44948	*ycf3_extron_2-ycf3_extron_1*	LSC	102081	*rps12-trnV-GAC*	IRA
48	P	39	44948	*ycf3_extron_2-ycf3_extron_1*	LSC	143691	*trnV-GAC-rps12*	IRB
49	P	36	8634	*psbI-trnS-GCU*	LSC	46681	*trnS-GGA*	LSC
*Epimedium_mikinorii*_GZLS								
1	P	131	5828	*trnK-UUU-rps16*	LSC	103584	*trnV-GAC-rrn16*	IRA
2	F	131	5828	*trnK-UUU-rps16*	LSC	141727	*rrn16*	IRB
3	F	107	4637	*trnK-UUU-rps16*	LSC	109586	*rrn23*	IRA
4	P	107	4637	*trnK-UUU-rps16*	LSC	135749	*rrn23*	IRB
5	F	82	113989	*ycf1*	IRA	114103	*ycf1*	IRA
6	P	82	113989	*ycf1*	IRA	131257	*ycf1*	IRB
7	P	82	114103	*ycf1*	IRA	131371	*ycf1*	IRB

8	F	82	131257	*ycf1*	IRB	131371	*ycf1*	IRB
9	P	71	62158	*accD-psaI*	LSC	62158	*accD-psaI*	LSC
10	F	77	4747	*trnK-UUU-rps16*	LSC	109698	*rrn23*	IRA
11	P	77	4747	*trnK-UUU-rps16*	LSC	135667	*rrn23*	IRB
12	F	62	131277	*ycf1*	IRB	131391	*ycf1*	IRB
13	F	53	4691	*trnK-UUU-rps16*	LSC	109640	*rrn23*	IRA
14	P	53	4691	*trnK-UUU-rps16*	LSC	135749	*rrn23*	IRB
15	P	48	54721	*trnV-UAC-trnM-CAU*	LSC	54721	*trnV-UAC-trnM-CAU*	LSC
16	F	53	113987	*ycf1*	IRA	114053	*ycf1*	IRA
17	P	53	113987	*ycf1*	IRA	131336	*ycf1*	IRB
18	P	53	114053	*ycf1*	IRA	131402	*ycf1*	IRB
19	F	53	131336	*ycf1*	IRB	131402	*ycf1*	IRB
20	F	49	4775	*trnK-UUU-rps16*	LSC	109726	*rrn23*	IRA
21	P	49	4775	*trnK-UUU-rps16*	LSC	135667	*rrn23*	IRB
22	P	49	98008	*trnL-CAA-ndhB*	IRA	98008	*trnL-CAA-ndhB*	IRA
23	F	49	98008	*trnL-CAA-ndhB*	IRA	147385	*ndhB-trnL-CAA*	IRB
24	P	49	147385	*ndhB-trnL-CAA*	IRB	147385	*ndhB-trnL-CAA*	IRB
25	P	44	29970	*petN-psbM*	LSC	29970	*petN-psbM*	LSC
26	F	47	72178	*rps12-clpP*	LSC	72223	*rps12-clpP*	LSC
27	F	50	74370	*clpP_extron_2-clpP_extron_1*	LSC	74418	*clpP_extron_2-clpP_extron_1*	LSC
28	F	46	114060	*ycf1*	IRA	114108	*ycf1*	IRA
29	P	46	114060	*ycf1*	IRA	131288	*ycf1*	IRB
30	P	46	114108	*ycf1*	IRA	131336	*ycf1*	IRB
31	F	46	131288	*ycf1*	IRB	131336	*ycf1*	IRB
32	F	37	92082	*ycf2*	IRA	92139	*ycf2*	IRA
33	P	37	92082	*ycf2*	IRA	153266	*ycf2*	IRB
34	P	37	92139	*ycf2*	IRA	153323	*ycf2*	IRB
35	F	37	153266	*ycf2*	IRB	153323	*ycf2*	IRB
36	F	40	131349	*ycf1*	IRB	131415	*ycf1*	IRB
37	P	36	36461	*psbC-trnS-UGA*	LSC	36509	*psbC-trnS-UGA*	LSC
38	P	35	5924	*trnK-UUU-rps16*	LSC	103584	*trnV-GAC-rrn16*	IRA

39	F	35	5924	*trnK-UUU-rps16*	LSC	141823	*rrn16-trnV-GAC*	IRB
40	P	35	65686	*petA-psbJ*	LSC	65728	*petA-psbJ*	LSC
41	P	43	49669	*trnL-UAA-trnF-GAA*	LSC	49669	*trnL-UAA-trnF-GAA*	LSC
42	F	43	113905	*ycf1*	IRA	113917	*ycf1*	IRA
43	P	43	113905	*ycf1*	IRA	131482	*ycf1*	IRB
44	P	43	113917	*ycf1*	IRA	131494	*ycf1*	IRB
45	F	43	131482	*ycf1*	IRB	131494	*ycf1*	IRB
46	F	33	131301	*ycf1*	IRB	131349	*ycf1*	IRB
47	F	39	44928	*ycf3_extron_2-ycf3_extron_1*	LSC	102025	*rps12-trnV-GAC*	IRA
48	P	39	44928	*ycf3_extron_2-ycf3_extron_1*	LSC	143378	*trnV-GAC-rps12*	IRB
49	P	36	8657	*psbI-trnS-GCU*	LSC	46664	*trnS-GGA*	LSC
*Epimedium_pseudowushanense*_GZNW								
1	P	131	5836	*trnK-UUU-rps16*	LSC	103606	*trnV-GAC-rrn16*	IRA
2	F	131	5836	*trnK-UUU-rps16*	LSC	141993	*rrn16*	IRB
3	F	107	4644	*trnK-UUU-rps16*	LSC	109608	*rrn23*	IRA
4	P	107	4644	*trnK-UUU-rps16*	LSC	136015	*rrn23*	IRB
5	F	82	114010	*ycf1*	IRA	114124	*ycf1*	IRA
6	P	82	114010	*ycf1*	IRA	131524	*ycf1*	IRB
7	P	82	114124	*ycf1*	IRA	131638	*ycf1*	IRB
8	F	82	131524	*ycf1*	IRB	131638	*ycf1*	IRB
9	P	71	62184	*accD-psaI*	LSC	62184	*accD-psaI*	LSC
10	F	77	4754	*trnK-UUU-rps16*	LSC	109720	*rrn23*	IRA
11	P	77	4754	*trnK-UUU-rps16*	LSC	135933	*rrn23*	IRB
12	F	62	131544	*ycf1*	IRB	131658	*ycf1*	IRB
13	F	53	4698	*trnK-UUU-rps16*	LSC	109662	*rrn23*	IRA
14	P	53	4698	*trnK-UUU-rps16*	LSC	136015	*rrn23*	IRB
15	P	48	54741	*trnV-UAC-trnM-CAU*	LSC	54741	*trnV-UAC-trnM-CAU*	LSC
16	F	47	92098	*ycf2*	IRA	92161	*ycf2*	IRA
17	P	47	92098	*ycf2*	IRA	153522	*ycf2*	IRB
18	P	47	92161	*ycf2*	IRA	153585	*ycf2*	IRB

19	F	47	153522	*ycf2*	IRB	153585	*ycf2*	IRB
20	F	53	114008	*ycf1*	IRA	114074	*ycf1*	IRA
21	P	53	114008	*ycf1*	IRA	131603	*ycf1*	IRB
22	P	53	114074	*ycf1*	IRA	131669	*ycf1*	IRB
23	F	53	131603	*ycf1*	IRB	131669	*ycf1*	IRB
24	F	49	4782	*trnK-UUU-rps16*	LSC	109748	*rrn23*	IRA
25	P	49	4782	*trnK-UUU-rps16*	LSC	135933	*rrn23*	IRB
26	P	49	98030	*trnL-CAA-ndhB*	IRA	98030	*trnL-CAA-ndhB*	IRA
27	F	49	98030	*trnL-CAA-ndhB*	IRA	147651	*ndhB-trnL-CAA*	IRB
28	P	49	147651	*ndhB-trnL-CAA*	IRB	147651	*ndhB-trnL-CAA*	IRB
29	P	44	29986	*petN-psbM*	LSC	29986	*petN-psbM*	LSC
30	F	47	72205	*rps12-clpP*	LSC	72250	*rps12-clpP*	LSC
31	F	50	74395	*clpP_extron_2-clpP_extron_1*	LSC	74443	*clpP_extron_2-clpP_extron_1*	LSC
32	F	46	114081	*ycf1*	IRA	114129	*ycf1*	IRA
33	P	46	114081	*ycf1*	IRA	131555	*ycf1*	IRB
34	P	46	114129	*ycf1*	IRA	131603	*ycf1*	IRB
35	F	46	131555	*ycf1*	IRB	131603	*ycf1*	IRB
36	F	40	131616	*ycf1*	IRB	131682	*ycf1*	IRB
37	P	35	5932	*trnK-UUU-rps16*	LSC	103606	*trnV-GAC-rrn16*	IRA
38	F	35	5932	*trnK-UUU-rps16*	LSC	142089	*rrn16-trnV-GAC*	IRB
39	P	35	65711	*petA-psbJ*	LSC	65753	*petA-psbJ*	LSC
40	P	43	49687	*trnL-UAA-trnF-GAA*	LSC	49687	*trnL-UAA-trnF-GAA*	LSC
41	F	43	113926	*ycf1*	IRA	113938	*ycf1*	IRA
42	P	43	113926	*ycf1*	IRA	131749	*ycf1*	IRB
43	P	43	113938	*ycf1*	IRA	131761	*ycf1*	IRB
44	F	43	131749	*ycf1*	IRB	131761	*ycf1*	IRB
45	F	33	131568	*ycf1*	IRB	131616	*ycf1*	IRB
46	F	39	44953	*ycf3_extron_2-ycf3_extron_1*	LSC	102047	*rps12-trnV-GAC*	IRA
47	P	39	44953	*ycf3_extron_2-ycf3_extron_1*	LSC	143644	*trnV-GAC-rps12*	IRB

48	P	36	8656	*psbI-trnS-GCU*	LSC	46689	*trnS-GGA*	LSC
49	F	32	5511	*trnK-UUU-rps16*	LSC	5662	*trnK-UUU-rps16*	LSC
Epimedium_ilicifolium_SXBX								
1	P	131	5827	*trnK-UUU-rps16*	LSC	103296	*trnV-GAC-rrn16*	IRA
2	F	131	5827	*trnK-UUU-rps16*	LSC	141644	*rrn16*	IRB
3	F	107	4634	*trnK-UUU-rps16*	LSC	109298	*rrn23*	IRA
4	P	107	4634	*trnK-UUU-rps16*	LSC	135666	*rrn23*	IRB
5	F	82	113701	*ycf1*	IRA	113815	*ycf1*	IRA
6	P	82	113701	*ycf1*	IRA	131174	*ycf1*	IRB
7	P	82	113815	*ycf1*	IRA	131288	*ycf1*	IRB
8	F	82	131174	*ycf1*	IRB	131288	*ycf1*	IRB
9	P	71	62201	*accD-psaI*	LSC	62201	*accD-psaI*	LSC
10	F	77	4744	*trnK-UUU-rps16*	LSC	109410	*rrn23*	IRA
11	P	77	4744	*trnK-UUU-rps16*	LSC	135584	*rrn23*	IRB
12	F	62	131194	*ycf1*	IRB	131308	*ycf1*	IRB
13	F	53	4688	*trnK-UUU-rps16*	LSC	109352	*rrn23*	IRA
14	P	53	4688	*trnK-UUU-rps16*	LSC	135666	*rrn23*	IRB
15	P	48	54725	*trnV-UAC-trnM-CAU*	LSC	54725	*trnV-UAC-trnM-CAU*	LSC
16	F	47	91777	*ycf2*	IRA	91840	*ycf2*	IRA
17	P	47	91777	*ycf2*	IRA	153184	*ycf2*	IRB
18	P	47	91840	*ycf2*	IRA	153247	*ycf2*	IRB
19	F	47	153184	*ycf2*	IRB	153247	*ycf2*	IRB
20	F	53	113699	*ycf1*	IRA	113765	*ycf1*	IRA
21	P	53	113699	*ycf1*	IRA	131253	*ycf1*	IRB
22	P	53	113765	*ycf1*	IRA	131319	*ycf1*	IRB
23	F	53	131253	*ycf1*	IRB	131319	*ycf1*	IRB
24	F	49	4772	*trnK-UUU-rps16*	LSC	109438	*rrn23*	IRA
25	P	49	4772	*trnK-UUU-rps16*	LSC	135584	*rrn23*	IRB
26	P	49	97714	*trnL-CAA-ndhB*	IRA	97714	*trnL-CAA-ndhB*	IRA
27	F	49	97714	*trnL-CAA-ndhB*	IRA	147308	*ndhB-trnL-CAA*	IRB
28	P	49	147308	*ndhB-trnL-CAA*	IRB	147308	*ndhB-trnL-CAA*	IRB
29	P	44	29960	*petN-psbM*	LSC	29960	*petN-psbM*	LSC

30	F	50	74362	*clpP_extron_2-clpP_extron_1*	LSC	74410	*clpP_extron_2-clpP_extron_1*	LSC
31	F	46	113772	*ycf1*	IRA	113820	*ycf1*	IRA
32	P	46	113772	*ycf1*	IRA	131205	*ycf1*	IRB
33	P	46	113820	*ycf1*	IRA	131253	*ycf1*	IRB
34	F	46	131205	*ycf1*	IRB	131253	*ycf1*	IRB
35	F	44	72179	*rps12-clpP*	LSC	72224	*rps12-clpP*	LSC
36	F	40	131266	*ycf1*	IRB	131332	*ycf1*	IRB
37	P	35	5923	*trnK-UUU-rps16*	LSC	103296	*trnV-GAC-rrn16*	IRA
38	F	35	5923	*trnK-UUU-rps16*	LSC	141740	*rrn16-trnV-GAC*	IRB
39	P	35	65722	*petA-psbJ*	LSC	65764	*petA-psbJ*	LSC
40	F	43	113617	*ycf1*	IRA	113629	*ycf1*	IRA
41	P	43	113617	*ycf1*	IRA	131399	*ycf1*	IRB
42	P	43	113629	*ycf1*	IRA	131411	*ycf1*	IRB
43	F	43	131399	*ycf1*	IRB	131411	*ycf1*	IRB
44	F	33	131218	*ycf1*	IRB	131266	*ycf1*	IRB
45	F	39	44950	*ycf3_extron_2-ycf3_extron_1*	LSC	101737	*rps12-trnV-GAC*	IRA
46	P	39	44950	*ycf3_extron_2-ycf3_extron_1*	LSC	143295	*trnV-GAC-rps12*	IRB
47	P	36	8639	*psbI-trnS-GCU*	LSC	46686	*trnS-GGA*	LSC
48	F	32	5502	*trnK-UUU-rps16*	LSC	5653	*trnK-UUU-rps16*	LSC
49	P	41	72175	*rps12-clpP*	LSC	107182	*trnA-UGC-rrn23*	IRA

Table S5. Voucher information and GenBank accession numbers for the *Epimedium* samples.

Section	Series	Latin name	Voucher No.	GenBank Assesion No.	SRA Assesion No.	Location	Sampling Part
Diphyllon	Brachycerae	*Epimedium borealiguizhouense*	GZQB	MK408751	SAMN12430115	Guiyang, Guizhou	Leaf
	Brachycerae	*Epimedium sagittatum*	/	KU204899	/	GenBank	/
	Brachycerae	*Epimedium dolichostemon*	/	KU522470	/	GenBank	/
	Dolichocerae	*Epimedium wushanense*	HBXS	MK408753	SAMN12430116	Xingshan, Hubei	Leaf
	Dolichocerae	*Epimedium wushanense*	HBXS-2	MK992920	SAMN12430117	Xingshan, Hubei	Leaf
	Dolichocerae	*Epimedium acuminatum*	/	KU522469	/	GenBank	/

	Dolichocerae	*Epimedium lishihchenii*	/	KU522472	/	GenBank	/
	Dolichocerae	*Epimedium chlorandrum*	SCMP	MK408754	SAMN12430118	Baoxing, Sichuan	Leaf
	Davidianae	*Epimedium mikinorii*	HBES	MK408752	SAMN12430119	Enshi, Hubei	Leaf
	Davidianae	*Epimedium mikinorii*	GZLS	MK992918	SAMN12430120	Leishan, Guizhou	Leaf
	Davidianae	*Epimedium ilicifolium*	SXBX	MK992921	SAMN12430121	Zhenping, Shaanxi	Leaf
	/	*Epimedium pseudowushanense*	GZJH	MK408750	SAMN12430122	Jianhe, Guizhou	Leaf
	/	*Epimedium pseudowushanense*	GZNW	MK992919	SAMN12430123	Guiyang, Guizhou	Leaf
	/	*Epimedium pseudowushanense*	/	KU522473	/	GenBank	/
Macroceras	/	*Epimedium koreanum*	/	KM207675	/	GenBank	/
	/	*Epimedium koreanum*	/	KU522471	/	GenBank	/

© 2019 by the authors. Licensee MDPI, Basel, Switzerland. This article is an open access article distributed under the terms and conditions of the Creative Commons Attribution (CC BY) license (http://creativecommons.org/licenses/by/4.0/).
